# A data-driven digital twin for water ultrafiltration

**DOI:** 10.1038/s44172-022-00023-6

**Published:** 2022-09-30

**Authors:** Jan Kloppenborg Møller, Goran Goranović, Per Brath, Henrik Madsen

**Affiliations:** 1 DTU Compute, Asmussens Allé Building 303B, Kgs. Lyngby, DK-2800 Denmark; 2 Grundfos Holding A/S, Poul Due Jensens vej 7, Bjerringbro, DK-8850 Denmark; 3 Present Address: Danfoss Drives A/S, Global R&D, Design Center Denmark, Pontoppidanstræde 101, Aalborg Ø, DK-9220 Denmark

**Keywords:** Chemical engineering, Scientific data, Environmental impact

## Abstract

Membrane-based separations are proven and useful industrial-scale technologies, suitable for automation. Digital twins are models of physical dynamical systems which continuously couple with data from a real world system to help understand and control performance. However, ultrafiltration and microfiltration membrane separation techniques lack a rigorous theoretical description due to the complex interactions and associated uncertainties. Here we report a digital-twin methodology called the Stochastic Greybox Modelling and Control (SGMC) that can account for random changes that occur during the separation processes and apply it to water ultrafiltration. In contrast to recent probabilistic approaches to digital twins, we use a physically intuitive formalism of stochastic differential equations to assess uncertainties and implement updates. We demonstrate the application of our digital twin model to control the filtration process and minimize the energy use under a fixed water volume in a membrane ultrafiltration of artificially simulated lakewater. The explicit modelling of uncertainties and the adaptable real-time control of stochastic physical states are particular strengths of SGMC, which makes it suited to real-world problems with inherent unknowns.

## Abstract (web summary)

Jan Kloppenborg Møller & Goran Goranović and colleagues introduce a data-driven twin methodology which balances physical knowledge with uncertainty quantifications. The approach makes it suited to application of real world problems with inherent unknowns. They demonstrate its application in the modelling and control of membrane water ultrafiltration

## Introduction

Membrane separation technologies are well established techniques of removal of unwanted particles from a solvent e.g. water, milk, wine, blood, fruit juice etc., categorized by the size of membrane pores: reverse osmosis (<1 nm), nanofiltration (1–2 nm), ultrafiltration (2–100 nm) and microfiltration (100 nm–10 μm)^1^. The common problem to all is that permeate flux through membranes diminishes due to particles that accumulate at the membrane surface (in the form of solid filtrate, or as concentration polarization of built-up salts), or penetrate and block the pores, both reversibly and irreversibly (the latter called membrane fouling). The systems are thus operated below a critical constant trans-membrane pressure to minimize the blockage and extend the lifetime of membranes^1,2^.

The ultra- & microfiltration lack rigorous theoretical description because of complex interactions and associated uncertainties including variable pore size and geometries, unknown surface forces of membranes, and nature of filtrate^3–11^. In current models of ultra- *&* microfiltration, Darcy’s linear phenomenological law (or its quadratic Darcy-Forchheimer extension for turbulent flows) is expanded by an extra resistance term to account for the filtrate^12^, making the flux vs. pressure dependence generally nonlinear. The flux’ decline is then usually modelled by an ordinary differential equation (ODE), expressing directly the flux’ differential change, with different power-law exponents of flux decay associated with different blocking mechanisms^12–15^.

Hydrodynamic boundary layer theory (based on partial differential equations, PDEs), both laminar and turbulent, describes the cross-flow versions of the above separation techniques, providing a spatial resolution^16^. The cross-flow transports away the accumulated particles or solutes at membrane’s surface thus increasing the permeate flux, Fig. S1a, Supplementary Note 1. Finally, various AI methods have been used to model (cross-flow) membrane filtrations^17^, including hybrid systems combining neural networks and physical theory^18^.

Digital twins present the latest stage of the models of physical dynamical systems, featuring a continual coupling between the virtual (modelling) and the physical domains of an experimental set-up^19^. Importantly, the critical component of a digital twin is singled-out to be the feedback (update) between the virtual and the physical domains,^20^, enabling (1) predictive control of the physical system^21^, and (2) the update of the virtual states of the system based on data^22^.

One problem of digital twins is identifying the right virtual models^22^. In general, one does not know beforehand the true model of a physical system—there can in fact be several virtual representations, each being a different yet good-enough model. Thus, a ranking of models is needed since one cannot assume a one-to-one mapping between the virtual and the physical domains so that the continual updating converges to the true model. Our digital twin approach addresses the issue by using stochastic differential equations (SDEs) for the models, differing from the approach of^22^. In addition to being physically intuitive, SDEs enable us to quantify model uncertainties (via diffusion terms). In fact, our methodology features actual modelling of the uncertainties to achieve the best fitting parameters from data for each proposed model. We then use two statistical measures to statistically rank the models.

The Stochastic Greybox Modelling and Control features two important novelties with respect to the above mentioned membrane separations theories, which together enable the online control: (1) we use time-dependent inputs (pressure and cross-flow, *P*(*t*) and *Q*(*t*)), which can be programmed to yield a particular outcome, say minimal energy use, Fig. S1a; and (2) we model in terms of the state variable(s)—here the thickness of the accumulated filtrate of which the flux is a function—pliable to control via *P*(*t*) and *Q*(*t*). The filtrate evolves stochastically via an SDE and affects the flux, and the control makes adjustments of the filtrate to achieve the desired flux. The filtrate is not directly measured but has to be reconstructed from the combined modelling and the flux measurements. The SDE quantifies various inherent uncertainties in the system and is able to accommodate the real-time random variations of the filtrate to make the optimal control corrections. For different models, there are different optimal solutions.

Our comprehensive article combines several disciplines (data science, physics, statistics, control theory, experimental design), but also couples theory to the experiments, a recognized need^23^. However, our main focus is on models—their build-up, validation and use for control purposes—in other words on data-driven future forecasting. The experiments and their analyses and interpretations, although useful, are less central and are thus placed in Supplementary Method 1 and Supplementary Discussion. We advise readers though, especially those coming from traditional membrane approaches, to read the Supplementary Methods 1 and 2 as a primer to the next section.

## Results and discussion

### Experimental design and data

The set-up, Fig. S1b, and the experiments of Supplementary Notes 1 and 2, are important in so far that they yield data: as unclean water (the recipe in Table S1) is passed through an ultrafiltration membrane in a controlled way via separate pressure (Δ*P*) and cross-flow (*Q*) pumps, a thick flow-retarding filtrate (cake) accumulates at the membrane, increasing with the pressure and diminishing with the cross-flow. The flux through the membrane is measured by weight. The idea is to perform the filtration with minimal energy during real-time operations.

Our digital twin is restricted to salient features of the filtration process, rather than featuring detailed computational fluid dynamics of the entire set-up, unsuitable for control purposes. Thus, the twin’s essential physical domain is^22^: measured native resistance of the membrane, unknown resistance of the accumulated filtrate, measured flux, and the time-dependent pressure and cross-flow inputs from programmable functioning pumps. The virtual domain is: data-driven updatable models of both the filtrate and the flux as a function of the filtrate, model parameters and statistical validation, and cost functions. The domains are coupled by the online control algorithms.

**Fig. 1:**
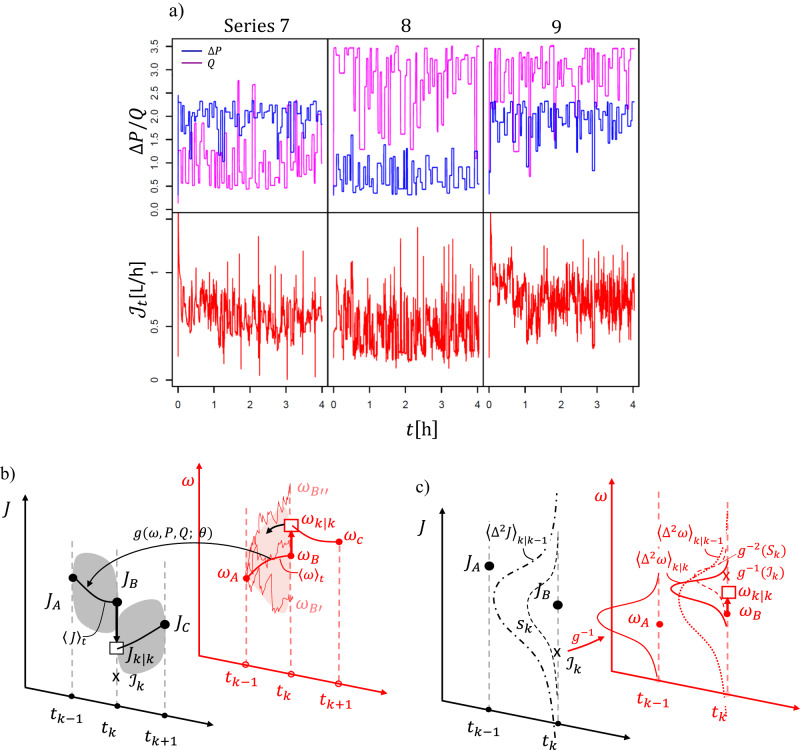
Data and the forecasting method. **a** The time-dependent input series 7, 8 and 9 (top) and their corresponding flux outputs (bottom). The input is randomized, but also programmable. The fluxes have two time-scales: the instantaneous one, corresponding to the abrupt changes in Δ*P* and *Q* (peaks), and a slower one, corresponding to the cake build-up (the downward trend). **b** Schematic of the Kalman filtering used for forecasting (prediction plus update). The predicted means (thick lines) of the hidden cake, 〈*ω*〉, and its function, the flux 〈*J*〉, along with their standard deviations (the shaded areas that replace the rugged Monte Carlo simulations); ${{{{{{{\mathcal{J}}}}}}}}$ are the flux measurements. The squares are the new values at time *t*_*k*_ updated with the measurements at *t*_*k*_; **c** The variances; minimizing the updated variance ${\langle {{{\Delta }}}^{2}\omega \rangle }_{k| k}$ determines the new position of the state, *ω*_*k*∣*k*_. The subscript _∣*k*_ indicates the update. For details see Methods, Filtering.

As mentioned, the pressure Δ*P*_*t*_ ≡ Δ*P*(*t*) and cross-flow *Q*_*t*_ are time dependent, and in fact rapidly varying in contrast to usual constant inputs in membrane science, Fig. 1a. This serves triple purpose. The first is the model identification: we statistically probe our system in a wide range of randomized input-output scenarios (23 in total) to identify model parameters to be valid across the entire range. Compared to traditional constant inputs the randomized inputs are statistically more reliable—the obtained model parameters are robust as both the choice and the number of data points is significantly larger, ~10^3^ − 10^4^, than in the case of traditional inputs, ~10. The accurate parameters are particularly important in data-driven models as ours, where the accent is to predict (as opposed to interpret) industrial operations that rely on limited processing time of data. Three randomized input series are shown in Fig. 1a (top row), and the rest are in Fig. S4a. Details of the randomized experimental design are given in Methods and further in Supplementary Note 3.

The second is control: our goal is the process control, subject to predefined constraints. That requires programmability of the input sequences, akin to the randomized variations. We will see in the Control section that cross-flow is indeed changing abruptly (counteracting the randomness of the filtrate) to achieve the minimal energy consumption. Hence, rapid time-dependent variations of the inputs paves the way for programming the inputs for any desired sequence—the crux of our digital-twin control.

And the third is time-resolved flux data: in Fig. 1a (bottom row), shown are flux measurements corresponding to the three Δ*P*_*t*_ and *Q*_*t*_ series. The striking feature of the data is the separation of time-scales, which are not discernible from the usual constant-input measurements. We see the instantaneous changes in the flux in response to the abrupt changes in the Δ*P*/*Q* (the sudden peaks in the fluxes of the series 7 and 8), as well as a slower, diffusive relaxation to the steady state related to the cake build-up (the flux of series 9). As known, the pressure changes propagate with the speed of sound, *c*^2^ = (∂*p*/∂*ρ*)_*s*_. The relaxation to the steady-state happens within a correlation time *τ*; for linear systems the decay is $\sim \exp (-t/\tau )$^24^. Our systems are non-linear and thus more complicated. Note that separate time scales are also present while reaching an equilibrium: a fast (pressure) vs. a slow (temperature) equilibration^25^.

The nearly instantaneous time scale provides justification for the Darcy’s law algebraic relation between the flux and the pressure. That is, the flux is a direct function of pressure and not given as a differential equation.

We note that a set of constant-input measurements were done prior to the randomization to adjust the level of appropriate fouling, Fig. S2b, Supplementary Note 2. Once the parameters are obtained, our models can of course predict for such inputs, Fig. S3.

### Stochastic greybox modelling

Stochastic greybox modelling combines physics with statistics and is mathematically involved^26–28^. The formalism is implemented in an R-package, CTSM-R (Continuous Time Stochastic Modelling for R)^29^, used in this study. Combining mechanistic understanding and statistical modelling will in general imply that the chosen models are simpler than what would be expected from a mechanistic point of view. Often, some effects are lumped in the description while model deficiencies are accounted by the stochastic diffusion terms. On the other hand, the statistical methods give a direct way of estimating parameters and quantifying uncertainties, both in terms of parameter uncertainties and prediction uncertainties.

For a given set of observations (time series) of flux ${{{{{{{{\mathcal{J}}}}}}}}}_{N}=[{{{{{{{{\mathcal{J}}}}}}}}}_{N},{{{{{{{{\mathcal{J}}}}}}}}}_{N-1},\ldots ,{{{{{{{{\mathcal{J}}}}}}}}}_{1},{{{{{{{{\mathcal{J}}}}}}}}}_{0}]$, we write the observation equation

1 $${{{{{{{{\mathcal{J}}}}}}}}}_{k}={J}_{k}+{e}_{k},$$

valid at discrete time points *t*_*k*_, *k* = 1, 2, …, *N*. ${{{{{{{{\mathcal{J}}}}}}}}}_{k}$ is the measurement and *J*_*k*_ the true value of the flux at *t*_*k*_, and *e*_*k*_ ~ *N*(0, *S*_*k*_) the (unknown) individual measurement error assumed to follow Gaussian distribution with expectation 0 and variance *S*_*k*_. We model the flux by time dependent Darcy’s law equation

2 $${J}_{t}=\frac{{{\Delta }}{P}_{t}}{{R}_{m}+{{R}_{c}}_{t}({\omega }_{t})}\equiv g({\omega }_{t},{{\Delta }}{P}_{t},{Q}_{t},t;\theta ),$$

where Δ*P*_*t*_ is time dependent pressure, *R*_*m*_ the constant native membrane resistance and ${{R}_{c}}_{t}$ the time-dependent extra resistance due to the cake formation. ${{R}_{c}}_{t}$ is a function of the hidden state *ω*_*t*_, the cake ‘thickness’. Note that the Darcy’s law of Eq. 2 is a particular choice of function *g*. *J*_*k*_ in Eq. 1 is the discrete value of *J*_*t*_.

The hidden state *ω*_*t*_, representing the model dynamics of the cake (or of some underlying physical phenomenon, in general) evolves by the following state equation, the SDE

3 $$d{\omega }_{t}=f({\omega }_{t},{{\Delta }}{P}_{t},{Q}_{t},t;\theta )dt+\tilde{{\sigma }_{t}}({\omega }_{t};\theta )d{W}_{t},$$

where *f* is commonly referred to as the drift term and $\tilde{\sigma }$ as the diffusion term. *f* is generally a complicated, non-linear function of its arguments (*θ* are parameters). $\tilde{\sigma }$ accounts not only for the physical diffusion, but also for the unknown aspects of the hidden state not captured by *f*, since the phenomenon’s true structure represented by *f* is often unidentifiable. *dW* is the differential Wiener process.

Eqs. 1–3 constitute our stochastic greybox framework.

The (extended) Kalman filtering^26,30,31^, used for the optimal updates of stochastic models with noisy data, and the maximum likelihood estimate, used to determine model parameters and to statistically validate the models, are expounded in details in Methods (Filtering, and Likelihood).

Here we briefly sketch the essence of the filtering through Fig. 1b, c, where the subscript _∣*k*_ denotes conditioning on measurement (‘given *k* measurements’, Supplementary Note 4). The stochastic state *ω*, cake thickness, is not directly measured and evolves continuously in time; it is predicted by a mean value and a variance from one time step to the next. The flux *J* is modelled as a function of *ω*. Upon the discreet measurement of flux in the current step, ${{{{{{{{\mathcal{J}}}}}}}}}_{k}$, the state *ω* is updated in the way that its variance in the current step conditioned on the measurements, ${\langle {{{\Delta }}}^{2}\omega \rangle }_{k| k}$ (the weighted sum of the state variance from the previous step and the measurement error of the current step), is minimized. That determines the updated value of the state, *ω*_*k*∣*k*_, and subsequently of the flux, *J*_*k*∣*k*_.

We point the reader to an instructive simple modelling example similar to the real models below, which illustrates the greybox approach and the use of CTSM-R (Continuous Time Stochastic Modelling for R) software, (Supplementary Note 5).

### The filtration models

Our models are modified (stochastic) versions of equations of the study^32^ (shown in Supplementary Note 6 for convenience. Also, our scaling and units differ from the literature; parameters are converted in Supplementary Note 7 and displayed in Tables S4 and S5), plus our own choices (*σ* and *J*_*ss*_ below). Our parametrization is:

- cake resistance *R*_*c*_(*ω*) (used in models: *M*1–*M*6)
  4 $${R}_{ct}=\left(1+\frac{{{\Delta }}{P}_{t}}{{P}_{a}({\omega }_{t},V)}\right){\omega }_{t},$$
  where *P*_*a*_ is a compressibility factor and *V* the total collected volume. Δ*P*_*t*_ is the time dependent pressure input.
- cake-thickness *ω* (the hidden state) (*M*1–*M*6)
  5 $$d{\omega }_{t}=\left({J}_{t}({\omega }_{t})-{J}_{ss}({Q}_{t})h({\omega }_{t})\right){c}_{b}dt+{\tilde{\sigma }}_{t}({\omega }_{t})d{W}_{t},$$
  models the stochastic evolution (build-up, break-up) of the cake. *J*_*ss*_ is the steady-state mean flux to which the system settles, dependent on the cross-flow *Q*_*t*_. *c*_*b*_ is the bulk concentration, and *h* a relaxation factor defined later. Eq. 5 for the state is a non-linear SDE with varying mean and the state-dependent diffusion, similar to the Ornstein-Uhlenbeck process^33^, Eq. S1 of the Supplementary Note 5. The state will revert to the mean value and attain a finite variance in the steady state. One of the aim of the modelling is to propose and test the functional relation *J*_*ss*_(*Q*), which is typically not obtainable directly from measurements^32^.
- diffusion $$\tilde{\sigma }$$ (*M*1–*M*6)
  6a $${\tilde{\sigma }}_{t}({\omega }_{t})={\omega }_{t}{\sigma }_{t},$$
  6b $${\sigma }_{t}={\sigma }_{0}{e}^{({\sigma }_{P}{{\Delta }}{P}_{t}+{\sigma }_{Q}{Q}_{t})},$$
  model the diffusive uncertainty in *ω*-space, the cake thickness. In the ordinary 3D space, particles with positive diffusion coefficient go in both positive and negative directions. The *ω*-space is strictly positive - there is no negative cake; also, no cake implies no diffusion, and larger cakes fluctuate more (more ways to break off/pile up). Hence we assume the diffusion coefficient $\tilde{\sigma }$ of the cake to depend linearly on the cake, Eq. 6a. With the help of Eq. 21b (on page 10) we get a guiding estimate of uncertainty
  7 $${\langle {{{\Delta }}}^{2}\omega \rangle }_{ss}\approx \frac{{\sigma }^{2}{\omega }^{2}}{-2A(\omega )},$$
  i.e., the steady-state variance depends on *ω* through both the diffusion term (~*ω*^2^) and the drift term *A*(*ω*) (the non-diffusive term of Eq. 5). A classic example where variance is explicitly calculated but not modelled is the stochastic damped oscillator^34,35^. The state dependence of $\tilde{\sigma }$ was mathematically resolved by separation of variables in the log domain, Methods, Lamperti. Finally, the relative diffusion *σ*_*t*_ is further assumed to depend on the input variables Δ*P*_*t*_ and *Q*_*t*_; this is to test if there are additional, implicit, uncertainty trends besides the one modelled with the linear-cake dependence.
- function *h* (*M*1–*M*6)
  8 $$h(\omega )=1-{e}^{-\frac{\omega }{{\omega }_{c}}},$$
  where *ω*_*c*_ is a relaxation factor.
- steady-state flux *J*_*ss*_
  9 $$M1\quad {J}_{ss}=const.,$$
  10 $$M2,5,6\quad {J}_{ss}={e}^{{\mu }_{0}+{\mu }_{1}Q+{\mu }_{2}{Q}^{2}},$$
  11 $$M3\quad {J}_{ss}=\frac{{e}^{{\mu }_{0}}}{1+{e}^{{\alpha }_{\mu }(Q-{Q}_{0})}},$$
  12 $$M4\quad {J}_{ss}={\mu }_{0}{Q}^{\gamma },$$
  model *J*_*ss*_ dependence on the cross-flow in four different ways: as a constant, an exponential polynomial, a switch function and a power-law function, Eqs. 9–12, respectively. The exponential dependence is a mathematical convenience to avoid non-physical results such as negative values of diffusion coefficients. Our models essentially differ in *J*_*ss*_. Note that Eqs. 9–12 are our guesses, the fact explored in the section Steady-state flux.

### Parameters and model validation

Parameter estimates and statistical validation of the models were done on all 23 data series, i.e. the 23 output series (flux) and the 23 pairs of input series (Δ*P*_*t*_ and *Q*_*t*_). There were in total 89 h of measurements sampled every 5 s, hence 89 ⋅ 3600/5 = 64000 data points for flux, pressure and cross-flow distributed over the 23 data series. All these points are used for statistical analysis. This exceeds substantially the ordinary measurements under constant pressure/cross-flow, which are on the order of 10 data points for the input data (e.g. a fixed pressure and a few variable cross-flows).

The parameters obtained from CTSM-R (Continuous Time Stochastic Modelling for R) are shown in Table 1 (and in SI units in Tables S4 and S5), written in statistical fashion: the mean value of each parameter spans across the models given as columns. Approximate 95% confidence intervals (±2 standard deviations) are given below it, in parentheses. Most parameters are quite well defined, with the exception of *ω*_*C*_ in models *M*4 − *M*6 where the presented Wald confidence intervals should not be trusted.

**Table 1:** Estimated parameters for the models; the means and the confidence intervals (±2 std).

	*M*1	*M*2	*M*3	*M*4	*M*5	*M*6
*ω*_0_	0.52	0.52	0.52	0.52	0.49	0.46
	(0.51; 0.54)	(0.51; 0.53)	(0.5; 0.53)	(0.51; 0.53)	(0.48; 0.51)	(0.45; 0.47)
*c*_*b*_	0.00092	0.00144	0.0016	0.00142	0.00136	0.00136
	(0.00082; 0.00104)	(0.00132; 0.00156)	(0.0015; 0.00171)	(0.00131; 0.00153)	(0.00132; 0.00153)	(0.00123; 0.00151)
*Pa*	11.6	11.62	11.58	11.6	*P*_*a*_(*V*)	*P*_*a*_(*ω*)
	(11.17; 12.04)	(11.2; 12.06)	(11.16; 12.02)	(11.18; 12.04)		
*J*_*ss*_	0.41	Parameters of Eq. 10	Parameters of Eq. 11	Parameters of Eq. 12	Parameters of Eq. 10	Parameters of Eq. 10
	(0.38; 0.44)					
*ω*_*C*_	0.019	0.019	0.019	0.019	0.019	0.019
	(0; 0.141)	(0; 0.11)	(0; 0.129)	(0; 2.66 ⋅ 10^11^)	(0; 1.29 ⋅ 10^11^)	(0; 4.90 ⋅ 10^8^)
*σ*_0_	0.0072	0.0072	0.0071	0.0072	0.0072	0.0071
	(0.007; 0.0074)	(0.007; 0.0074)	(0.0069; 0.0073)	(0.007; 0.0074)	(0.007; 0.0074)	(0.0069; 0.0073)
*σ*_*P*_	0.116	0.105	0.105	0.106	0.104	0.207
	(0.101; 0.131)	(0.09; 0.119)	(0.091; 0.12)	(0.092; 0.121)	(0.09; 0.119)	(0.191; 0.223)
*σ*_*Q*_	−0.207	−0.196	−0.191	−0.196	−0.196	−0.204
	(−0.22; −0.2)	(−0.21; −0.19)	(−0.2; −0.18)	(−0.22; −0.2)	(−0.21; −0.19)	(−0.2; −0.18)
*df*	10	12	12	11	13	13
AIC	−520710	−521204	−521298	−521126	−521400	−521878
RMSE	0.176	0.137	0.135	0.138	0.133	0.130

For the values in SI units see Table S5. The last three lines give total number of parameters, or degrees of freedom (*df*), the Akaike Information Criterion (AIC) for each model and the pointwise distance between unconditional predictions and observations of the flux, i.e. the root mean square error (RMSE).

Physically, the filtrate is slightly compressible (~20%; *P*_*a*_ ~ 10), a part of it quickly formed (*ω*_0_ ≡ *ω*_*t*=0_ ≠ 0), and there are extra Δ*P* and *Q* contributions on diffusion, accounted by non-zero *σ*_*P*_ and *σ*_*Q*_. For more comments on the parameters see Supplementary Note 8.

Akaike Information Criterion (AIC) and the root mean square error (RMSE) statistically rank the models in Table 1 (defined in Methods, Likelihood).

### Model predictions vs. experiments

**Fig. 2:**
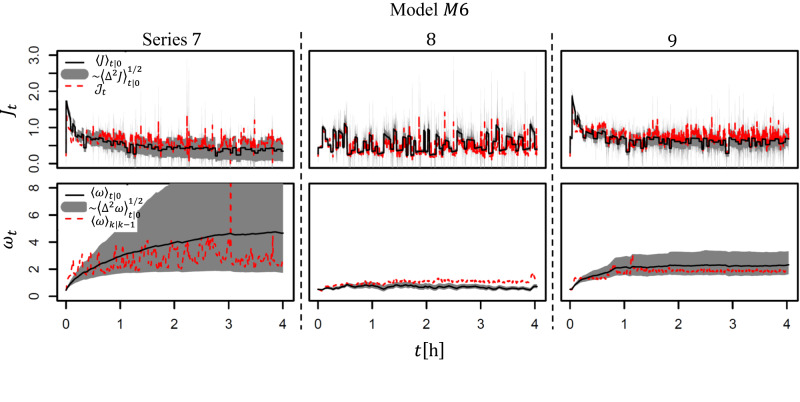
Experiments (ser. 7, 8 and 9) vs. model *M*6: fluxes (top) and cakes (bottom). ${{{{{{{{\mathcal{J}}}}}}}}}_{t}$ and 〈*ω*〉_*k*∣*k*−1_ are within the 95.4% prediction intervals (grey areas) for series 7 and 9, but outside them for series 8. Note the difference in prediction intervals of the cake/filtrate for the three series, depending on the interplay between Δ*P* and *Q*. See text for details.

In Fig. 2 we test the experimental series 7, 8 and 9 of Fig. 1a against the best model *M*6. Here it will be useful to relate to Fig. 1b (and Fig. S5 of the example), and Table S2 for nomenclature. The three series contain various characteristic features such as the variable sizes of the prediction intervals of both flux and cake, and reconstructed cake estimates. The analysis will help interpret other series (see later Figs. S6–S12, Supplementary Note 9).

The top row of Fig. 2 features the fluxes: the measured ${{{{{{{{\mathcal{J}}}}}}}}}_{t}$ (in red) vs. the long-term mean 〈*J*〉_*t*∣0_ (the black line) and its prediction interval $\pm 2\sqrt{{\langle {{{\Delta }}}^{2}J\rangle }_{t| 0}}$ (two standard deviations, in grey). The bottom row features the time evolution of the underlying cake thicknesses. Here the long-term predictions are ${\langle \omega \rangle }_{t| 0}\pm 2\sqrt{{\langle {{{\Delta }}}^{2}\omega \rangle }_{t| 0}}$. Because the cake is not directly measured, the red line here is the one-step ahead prediction 〈*ω*〉_*k*∣*k*−1_ (or 〈*ω*〉_*t*∣*t*−1_ for continuous *t*), the best estimate of the actual cake in the absence of its measurement.

In series 7 and 9, we see that ${{{{{{{{\mathcal{J}}}}}}}}}_{t}$ fall within the prediction intervals of the *M*6 (note the grey spikes modelling the red ones), the model thus being an appropriate description. The same series show the exponential relaxation of the flux at the beginning towards a steady value, as the cake builds up. In series 8, particularly in the second half, the measurements are out of the grey prediction intervals, hence *M*6 does not fit the series that well. Note that the mean flux predictions 〈*J*〉_*t*∣0_ as well as the prediction interval’s edges are uneven, owing to the time dependent input.

The mean predicted value of the cake 〈*ω*〉_*t*∣0_ (black line) is the largest in ser. 7 and the smallest in ser. 8 where it is almost completely removed by the cross-flow. The reason is the input series: low $\bar{Q}$, mid $\overline{{{\Delta }}P}$ (ser. 7); high $\bar{Q}$, low $\overline{{{\Delta }}P}$ (ser. 8) and high $\bar{Q}$ mid, $\overline{{{\Delta }}P}$ (ser. 9), Fig. 1a. We remind that the red line here represents the theoretical reconstruction of the cake, 〈*ω*〉_*k*∣*k*−1_, updated on the flux measurements (the closest one gets to the unobservable cake), rather than the cake measurements themselves, as in Fig. 1b. We infer that the cake oscillates wildly in series 7, in sync with the cross-flow input, but much less so in ser. 8 and 9. In ser. 8 the model predicts too large cake’s removal, underestimating the cake’s (reconstructed) thickness. In ser. 9 the cake reaches a steady state.

The cake’s 95% prediction intervals seem very large for ser. 7. The mathematical reason is our model for uncertainty, Eq. 6a, making the variance large, Eq. 7. Physically, this pertains to the case of the ordinary diffusion coefficient not being a constant but a function of the cake thickness ($D\equiv 1/2\,{\tilde{\sigma }}^{2}=D({\omega }^{2})$). The analogous concentration dependency of the diffusion coefficient *D*(*c*^2^) can indeed be obtained in ultrafiltration,^2^. Hence, our diffusion model is not unrealistic. Besides, the uncertainties also reflect the variations within the batch of the membranes.

Experimental findings of Supplementary Note 2 likely point to both irreversible and reversible parts of the filtrate, i.e. to a thin hardened cake that had to be removed chemically, and an embedded concentration polarization of the salts (particularly CaCl_2_ hydrates), respectively. Both of the phenomena are known to occur in ultrafiltration,^2^. It is the reversible parts that are probably being affected by the input in ser. 7 causing the filtrate’s oscillations. Thus, flux decays through an increased cake resistance and a fluctuating osmotic pressure. Our models are unaffected by the mechanisms though, as both contributions are implicitly accounted in the Darcy’s resistance *R*_*ct*_, as shown in e.g.^12^.

Note from Fig. 2 that the large cake’s prediction interval of ser. 7 does not result in as large flux’ prediction interval. The mathematical reason is that the flux variance depends as ~ 1/*ω*^4^, Eq. 19b (see *C* below Eq. 21b). The physical reason is the known phenomenon of permeate flux reaching a constant value independent of applied pressure as a large cake/gel forms (the limiting or critical flux). The system becomes mass transfer dependent and adjusts the cake thickness in response to pressure changes, leaving the flux essentially unchanged^2^. From Fig. 2, the fluxes yield a much narrower range of values, up to ~ 0.7 [L h^−1^].

Lastly, we report a few general trends and a couple of deficiencies. By inspecting the cake/CP filtrates across all 23 series in Figs. S7–S12 against their inputs in Fig. S4a, we notice as in Fig. 2 that the filtrates as well as their prediction intervals decrease at higher $\bar{Q}$ and lower $\overline{{{\Delta }}P}$ (ser. 8, beginnings of ser. 16 and 18), and increase in the opposite situation, at lower $\bar{Q}$ and higher $\overline{{{\Delta }}P}$ (ser. 7, middle of ser. 16 and 19). There are frequent variations in the filtrate thicknesses for ser. 1–5, due to rapid changes of concurrent high $\bar{Q}$ and high $\overline{{{\Delta }}P}$. Filtrate grows step-wise in ser. 22, in sync with the increasing Δ*P* and decreasing *Q*.

**Fig. 3:**
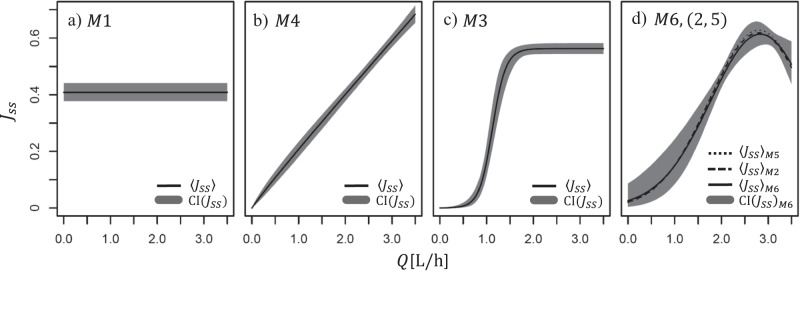
Functional dependencies of the steady-state flux, *J*_*ss*_(*Q*) (Eqs. 9–12 with parameters of Table 1). **a** A constant function *J*_*ss*_ = 0.41 [L h^−1^] of *M*1 is the simplest and the least accurate, representing an average *J*_*ss*_ across all 23 series; **b** power-law dependence *J*_*ss*_ ~ *Q*^*γ*^ of *M*4 encompassing *γ* = 1, i.e. a simple linear relationship *J*_*ss*_ = 0.2*Q*, within the confidence intervals around the obtained mean *γ* = 0.98. **c** Logistic function of *M*3 providing a critical-flux plateau above *Q* ≃ 1.5[L h^−1^], and **d** exponential polynomial of models *M*2, *M*5 and *M*6, statistically the most accurate. The functions of models *M*2-6 gave large statistical improvements relative to *M*1 (CI confidence intervals).

Ser. 8 and 6 feature opposite cross-flow inputs, i.e. high and low $\bar{Q}$, respectively (Figs. 1a and S4a). From Figs. S 7–S 12, all models *M*2-6 underestimate the reconstructed cake in ser. 8 but correctly predict ser. 6; *M*1 does the opposite: predicts well ser. 8 but underestimates ser. 6. The reason is the nature of models, Fig. 3. *M*1 gives a constant value of steady-state flux *J*_*ss*_(*Q*), i.e. an average *J*_*ss*_(*Q*) for all series. Good at high *Q* (ser. 8), the average overshoots *J*_*ss*_ and thus the cake removal at low *Q* (ser. 6); *M*2-6 do the opposite, perform well at low, but overshoot at high *Q*.

None of the models is perfect, hence the statistical ranking. The fact that a single series is not predicted correctly (within confidence intervals) by a model, corresponds to a single point outlier, say from a linear law/graph, in traditional single constant-input measurements. With the complex interactions of many different molecular species (Supplementary Note 2), a theoretical mismatch is inevitable.

### Steady-state flux *J*_*ss*_(*Q*)

Model Eqs. 9–12 represent different functional dependencies of the steady output flux on the cross-flow, *J*_*ss*_(*Q*), and are plotted in Fig. 3. The plots are useful since the dependence is typically not deducible directly from (few) measurements^32^.

Eq. 9 is a baseline model *M*1, and Eqs. 10–12 different generalizations of it: Eq. 10 (*M*2, 5, 6) is quite flexible but the parametrization imply that it is strictly positive (parameters *μ*_1_ = *μ*_2_ = 0 recover Eq. 9); Eq. 11 (*M*3) is monotone and reaches a maximum at some level of *Q* (*α*_*μ*_ = 0 recovers the baseline model), and finally Eq. 12 (*M*4) is monotone but less flexible than the two other models. All the suggested models give large improvements compared to the baseline in both the likelihood (measured by AIC), as well as in predictive power, measured as average distance (RMSE) between predicted values and observations (not using filtering), Table 1. *M*6 is the best giving the lowest AIC and RMSE values.

The steady-state is the result of the mass-balance between the convective and back-diffusive fluxes yielding the unchanging cake thicknesses and constant permeate fluxes^3^. The maximum value of the steady-state flux in ultrafiltration is the earlier mentioned critical flux; as said, it remains constant when pressure is increased beyond a certain value as any further increase in the pressure gets compensated by cake/gel thickening that increases resistance and lowers the flux back to the initial point^2^. When irreversible component exists, as in our system, one expects that the flux would be insensitive to cross-flow as well.

The models of Fig. 3 predict *J*_*ss*,m*ax*_ ≡ *J*_crit_ ~ 0.41 − 0.7 [L h^−1^]. Models *M*_1_ and *M*_3_ in addition predict a range of constant plateau values where *J*_*ss*_(*Q*) does not change (with *M*1 giving an overall average value thus being the least accurate). Statistically, the advantage is with model *M*6 with *J*_crit_(*Q*) ≃ 0.65 [L h^−1^], presumably reflecting the complicated nature of the filtrate.

*J*_*ss*_ depends also on pressure, but we limited our already detailed analysis to suit cross-flow based control. The pressure effects are partly lumped into non-zero *σ*_*P*_ and limit the flux’ range as discussed in the previous section in connection to prediction intervals of ser. 7.

We conclude that ‘the correct’ virtual model is determined in relative and not absolute terms. It was thus important that the statistical experimental design probed the system over a wide range of input values, leading to reliable model parameters. Each model can be programmed for control scenarios, but the more accurate models will effect desired cost functions more precisely under a random realization.

### Control strategies

In this section we minimize the energy primarily consumed by the cake-controlling cross-flow, under the constraint of obtaining a fixed volume of water. Such a scenario could be relevant in preexisting industrial operations where delivery of fixed amount of filtered solvent needs to be automated under minimal cost.

The control depends on three factors: (1) state-space formulation that enables control of the state, Eq. 18a, and thus the observable Eq. 2, (2) the Kalman filtering that enables updates with data, Eq. 20a, and thus corrections of predicted states, and (3) time-dependent input Δ*P*_*t*_ and *Q*_*t*_ which can be programmed to yield a desired outcome.

Our approach to control the underlying stochastic state (cake), differs from the approaches that include backwashing process, e.g., Ref. ^36^, or employ neural networks^37^. It is similar to study^38^, and is to our knowledge the first in the context of membrane ultrafiltration.

In the present work it was not possible to finalize online control on the real physical system, so we illustrate the principle by a realistic simulation in which the cake’s randomness is modelled by the variance $\tilde{\sigma }$ obtained from the data fitting, Table 1. We use the model *M*3 as it is easier (for experimentalists) to physically interpret it.

The control problem is

13a $$\min \int\nolimits_{0}^{T}S({{\Delta }}{P}_{t},{Q}_{t})dt;$$

13b $$\left\langle \int\nolimits_{0}^{T}{J}_{t}({{\Delta }}{P}_{t},{Q}_{t},t)dt\right\rangle ={V}_{0},$$

where we want to find (Δ*P*_*t*_, *Q*_*t*_) that minimize the integral of the loss function *S*( ⋅ ), under the constraint of the total expected volume from the model equations equalling the predefined volume *V*_0_. The loss function is chosen as

14 $$S({Q}_{t})=\int\nolimits_{t}^{T}{Q}_{t}^{3}dt$$

since the main contribution of energy loss was associated with the pump regulating the cross-flow, its energy proportional to cross-flow cubed (*E* ~ Δ*pQ* ~ *ρv*^2^*v* ~ *v*^3^). In general, pressure regulation also contributes to energy loss, but this was a smaller contribution in our test trials, Fig. S13 in Supplementary Note 10, and is easily accommodated into Eq. 14.

Technically, Δ*P*_*t*_ and *Q*_*t*_ are expanded into orthogonal (Legendre) polynomials and then the coefficients of the expansion are found which satisfy the above constraint; Δ*P*_*t*_ and *Q*_*t*_ are further constrained in range, see Methods, Expansion.

In our first control scenario, the fixed control, Δ*P*_*t*_ and *Q*_*t*_ are fixed at the beginning and not updated with time. We want to see which optimal control yields an average of 3 L, on the time horizon of 4 h (the length of experiments). The constraint, Eq. 13b, is included into the objective function, Eq. 13a, by

15 $$\min \left[\int\nolimits_{0}^{T}{Q}_{t}^{3}dt + \lambda {\left(\left\langle \int\nolimits_{0}^{T}{J}_{t}({Q}_{t},{{\Delta }}{P}_{t},t)dt\right\rangle -{V}_{0}\right)}^{2}\right],$$

where the Lagrange multiplier *λ* is the penalty parameter ensuring that the integral does not veer off the target value *V*_0_. *λ* is tuned by trial and error (~100). Putting the equations and the parameters from the model *M*3 and the expansions from Eqs. 31–33b into Eq. 15, one can solve for the optimal expansion coefficients using any general purpose optimizer algorithm e.g. found in R software.

**Fig. 4:**
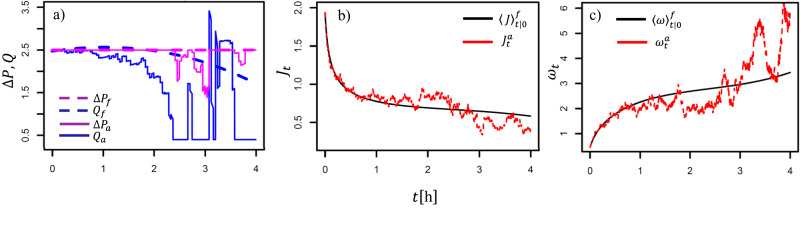
Two control strategies: fixed, *f*, without updates during *T* = 4 h, and adaptive (updated), *a*, with updates every *t*_*k*_ = 2.5 min; **a** the fixed and updated inputs (dashed/full lines) for pressure (magenta) and cross-flow (blue). **b** The optimized average flux based on the fixed control (black), and the optimized realized flux based on the updated control (red). **c** The optimized average cake dynamics based on the fixed control (black) and the optimized realized cake dynamics based on the updated control (red). The realized variables are on realistically simulated data (the cake variance based on fitting).

The optimizer gives a constant (highest possible) Δ*P* and a high *Q* that diminishes towards the end of the time interval, Fig. 4a dashed lines. Under this control, the resulting flux 〈*J*〉_*t*∣0_ and the corresponding cake 〈*ω*〉_*t*∣0_ are given by the black lines in panels b and c. Note the steady build-up of the cake as the cross-flow dwindles. The area below 〈*J*〉_*t*∣0_ is equal to the total collected volume of water, i.e. ∫〈*J*〉_*t*∣0_*dt* = 3.

Note that we have used the long-term predictions—the mean values 〈*ω*〉_*t*∣0_ and 〈*J*〉_*t*∣0_—to get the control that provides desired average behaviour of the cake and the flux during the 4 h period.

Our second scenario is the adaptive control: on shorter time scale the flux exhibits random fluctuations away from the anticipated average value that satisfies the constraint, hence corrections must be made. Say that at time *t*_*k*_ we have collected a total volume *V*_*k*_; *V*_*k*_ is now subtracted from the target *V*_0_ in the updated objective function

16 $$\min \left[\int\nolimits_{{t}_{k}}^{T}{Q}_{t}^{3}dt + \lambda {\left(\left\langle \int\nolimits_{{t}_{k}}^{T}{J}_{t}({Q}_{t},{{\Delta }}{P}_{t},t)dt\right\rangle -({V}_{0}-{V}_{k})\right)}^{2}\right],$$

and a new optimal strategy calculated. This step is repeated at any further *t*_*k*_, effectively re-applying Legendre polynomials to Δ*P*_*t*_ and *Q*_*t*_ for the remaining time horizon. The series of optimization problems results in the series of newly obtained (updated) expansion coefficients. In our case, *t*_*k*_ = 2.5 min.

For our stochastic realization, the updated Δ*P*_*t*_ and *Q*_*t*_ are shown by the full lines in Fig. 4a. The *Q*_*t*_ drifts downwards meaning that the realized flux is higher than the anticipated 〈*J*〉_*t*∣0_, so the control tries to lessen the removal of the cake (between 1–3 h; compare with the flux and the cake in (b) and (c), in red); around 3 h, *Q*_*t*_ is suddenly increased to compensate as the flux veers off lower than anticipated; *Q*_*t*_ also goes flat in three instances as it reaches *Q*_*min*_ set by Eq. 33a. Δ*P*_*t*_ remains a high constant except when *Q*_*t*_ = *Q*_*min*_.

Note that the close up of the adaptive (updated) ${J}_{t}^{a}$ and ${\omega }_{t}^{a}$ is that of Fig. 1b, with shifts due to updates, and hence different from what the stochastic realization would have been without the control (Fig. S5c). The adaptive control also makes $\int {J}_{t}^{a}dt=3$.

Incidentally, two of our series actually produce very close to 3 L during the 4 h test periods: series 9 and 20. We can thus compare the controls with two real life experiments producing the same quantity of water, Table 2. From the last table column, we see that the Δ*P*_*t*_ and *Q*_*t*_ sequences of the series 9 and 20 use more energy than the two control schemes.

**Table 2:** Comparison of the optimal controls with two experimental series.

	*V*	$\overline{{{\Delta }}P}$	$\bar{Q}$	∫*Q*^3^
Fixed ctrl.	3.00	2.50	2.35	53.74
Adaptive ctrl.	2.97	2.42	1.73	35.64
Ser. 9	3.02	1.93	2.86	105.40
Ser. 20	2.96	1.72	2.45	69.08

*V*—collected volume (integrated flux), $\overline{{{\Delta }}P}$—average pressure, $\bar{Q}$—average cross-flow, ∫*Q*^3^—consumed energy.

Adaptive control is the most efficient of the four, having the smallest average $\bar{Q}$. Compared to series 9, the adaptive control uses 66% less energy. Not all stochastic realizations, though, will yield such savings. Compared to the initially given fixed control, the updating apparently gives a higher flexibility.

## Conclusions

Stochastic Greybox Modelling and Control is a digital-twin methodology that uses stochastic differential equations (SDEs). SGMC predicts mean values and variances of (hidden) physical states, given the uncertain observations of functions of these states; it reconstructs the states, based on data, and is able to control them under desired constraints; it also provides statistical measures to quantify the merits of virtual models. A key aspect is the time-dependence of input variables, which enables their programmability. Randomized input parameters help identify models, but are not a necessity in general.

While the greybox models can in principle be used in any situation where a set of ODEs describe the phenomena at hand, the approach works best for reduced order models (possibly lumped) i.e. models where the deterministic part (drift term) of the stochastic differential equation describes only the most important phenomena, while the stochastic part (the diffusion term) then takes care of the deviations from the deterministic part, i.e. model approximations, measurement errors for the input/forcing variables and unrecognized input variables. Besides, there can be computational concerns in very high-dimensional problems, such as weather systems, where estimating the likelihood for the model parameters is rather computationally intensive. This can hinder early identification of models for timely concurrent control.

As in any statistical modelling, overfitting can pose a problem. Including more model parameters on training data does not guarantee a better score on independent data. One typically cross-validates the sets (cyclically removes one set and predicts for it based on the others), as in Ref. ^39^. We did not attempt that here as it was not our focus.

SGMC works with time-series and does not provide spatial resolution, as e.g. computational fluid dynamics simulations do. It strength lies in quantifying the uncertainties in time-series for optimal control purposes. Apportioning of uncertainties, conditioning on data and statistical validation makes the method mathematically involved. Once implemented, however, aspects of SGMC are proving useful in real-world settings, such as waste-water treatment plants or wind-energy production^38,40,41^.

## Methods

### Experimental design

The original data had a sampling frequency of 1 Hz (every second), but we lowered it to 0.2 Hz (every 5 s) for easier handling by taking the average over 5 s intervals. This was mainly to prevent the instances of zero permeate flux as there were hardly any drops of water passing through membrane over very short times, requiring a more complicated statistical analysis. The averaging does not affect our conclusions, as the time resolution is sufficient to distinguish the instantaneous and the diffusive time scales of the flux as well as programmable changes in the input series, Fig. 1a.

The pressure ranged between (0.5,3) bar, and the cross-flow between (0.5,3.5) [L h^−1^]. The series 1–10, 11–20 and 21–23 are distinguished by temporal changes in the range of 1/10, 1/4 and 1/2 of an hour, respectively. See Fig. 1a (top of the panel) and S4a.

There were two parameters to be randomized in the data series: the time between shifts and the actual values of pressures and cross-flows. The pressure and cross-flow variables were randomized independently.

We used two beta distributions to randomly control the time shifts, Fig. S4b. For the series 1–10, the time between shifts is drawn as an (independent) random number from a gamma distribution with parameter *α* = 6 and $\beta =\frac{6}{230}$, Fig. S4b, black line (since the aim of the modelling was long-term predictions, the designed time between shifts is long compared with the time constant from data so that the system ideally reaches a steady state every time. Here, the average time between shift was chosen as 230 s i.e. three times the time constant determined by a fit on data from pilot experiments).

For the series 11–20, the average time between shifts was changed to 460 s, and the time between shifts was drawn from a gamma distribution with parameter *α* = 12 and $\beta =\frac{12}{460}$, Fig. S4b, red line (by inspecting results of series 1–5, the system did not seem to settle to a steady state before a new shift, hence the extension).

With respect to Δ*P* and *Q* values, the following distributions were used.

Series 1 was designed to span different situations by drawing from a distribution proportional to the sum of the distances to all points that were visited previously by the experiment (Δ*P* and *Q* are treated independently). Series 2–5 are designed to span the space locally (in time); the distributions are defined in the same way as series 1, but only local observations are considered (defined by the time to the next shift). The 1–5 series was adapted manually to avoid fast fouling. This adaptation was done by changing cross flow below 0.2 m^3^/h to 0.2 m^3^/h and one below 0.4 m^3^/h to 0.4 m^3^/h when pressure was above 1 bar (series 1–3), or above 2.3 bar (series 4–5).

The series 6–9 were designed to span the space of the inputs such that the bias of each series is (series*#*: Δ*P*, *Q*): (6: low, low), (7: high, low), (8: low, high), (9: high, low). In practice this is done by drawing from beta-distributions (modified to favour large shifts). Series 10 is closer to the centre of the allowed inputs (also ensured by the modified beta-distributions). The manual adaptations of Δ*P* and *Q* were the same as for series 1–5.

The design of Series 11–20 was also based on modified beta-distributions: Series 11–16 are designed to complete one loop of different situations (e.g. going from low pressure to high pressure and back again), and series 17–20 to ensure that the entire phase space is spanned.

### Filtering: predictions and updates

Schematic of the Kalman filtering, the process of enabling time-series forecasting by combining models with actual measurements, is shown in Fig. 1b, c. In panel b, the observable *J* (flux) and its corresponding hidden state *ω* (cake thickness) are shown in our novel dual graph representation. *ω*_*B*,*C*_ and *J*_*B*,*C*_ are the one-step ahead predictions i.e. *ω*_*B*_ = *ω*_*k*∣*k*−1_ and *ω*_*C*_ = *ω*_*k*+1∣*k*_, *J*_*B*_ = *J*_*k*∣*k*−1_ and *J*_*C*_ = *J*_*k*+1∣*k*_; ${{{{{{{{\mathcal{J}}}}}}}}}_{k}$ are the measurements of flux at time *t*_*k*_, and *ω*_*A*_, *ω*_*k*∣*k*_ and *J*_*A*_, *J*_*k*∣*k*_ are the updated values. The subscript _∣*k*_ indicates the conditioning on *k* previous measurements; if there is no measurement updates, the symbol is _∣0_ i.e. long-term prediction, Table S2.

The cake *ω* is a random variable, but instead of computing its individual Monte Carlo realizations (jagged lines from *ω*_*A*_ to ${\omega }_{B^{\prime} }$, *ω*_*B″*_ etc.), the mean value 〈*ω*〉 and the variance ${\langle {{{\Delta }}}^{2}\omega \rangle }_{k| k-1}$ are computed; 〈*ω*〉 is obtained via *f*, i.e. integrating Eq. 3, and translated via *g* into 〈*J*〉, Eq. 2. The update with measurements ${{{{{{{{\mathcal{J}}}}}}}}}_{k}$, shifts *ω*_*B*_ to *ω*_*k*∣*k*_ and consequently *J*_*B*_ to *J*_*k*∣*k*_; *ω*_*k*∣*k*_ and *J*_*k*∣*k*_ become new initial points for the next one-step ahead predictions *ω*_*C*_ = *ω*_*k*+1∣*k*_ and *J*_*C*_ = *J*_*k*+1∣*k*_. Note that to calculate *ω*_*C*_ = *ω*_*k*+1∣*k*_ one must effect the *k* updates: *ω*_*k*∣*k*_, *ω*_*A*_ and all the earlier ones. The symbol _∣*k*−1_, “given *k* − 1”, thus tags that *k* − 1 previous updates have been made. Different models correspond to different functions *f* and *g*. Note that above and in Fig. 1b, c, conditioned variables are written as e.g. *ω*_*k*∣*k*_ and *J*_*k*∣*k*_ instead of 〈*ω*〉_*k*∣*k*_ and 〈*J*〉_*k*∣*k*_, for easier following.

In panel c shown are the variances. The model-predicted variance of the cake, ${\langle {{{\Delta }}}^{2}\omega \rangle }_{k| k-1}$ (dotted red line), is a function of $\tilde{\sigma }$ and quantifies dispersion of Monte Carlo realizations in the step *k*. The corresponding one-step ahead flux variance ${\langle {{{\Delta }}}^{2}J\rangle }_{k| k-1}$ (dot-dash black line) includes also the measurement error *S*_*k*_ (dashed black line). Both $\tilde{\sigma }$ and *S*_*k*_ are unknown and determined from data in the overall parameter optimization process. Upon the data update, the updated variance ${\langle {{{\Delta }}}^{2}\omega \rangle }_{k| k}$ (thick red line) becomes smaller than ${\langle {{{\Delta }}}^{2}\omega \rangle }_{k| k-1}$; in fact, it becomes minimal, and the minimization condition determines the optimal position of *ω*_*k*∣*k*_.

Formally, when *f* and *g* depend linearly on the state *ω* and the input *u* we have

17a $$f=A{\omega }_{t}+B{u}_{t},$$

17b $$g=C{\omega }_{t}+D{u}_{t},$$

where *A*, *B*, *C* and *D* are a subset of unknown fitting parameters *θ* (*A* < 0 for stability reasons). With *θ* determined, the state prediction equations of the linear Kalman filter are^5^

18a $$\frac{d{\langle \omega \rangle }_{t| k}}{dt}=f\left({\langle \omega \rangle }_{t| k}\ldots \right)=A{\langle \omega \rangle }_{t| k}+B{u}_{t},$$

18b $$\frac{d{\langle {{{\Delta }}}^{2}\omega \rangle }_{t| k}}{dt}=2A{\langle {{{\Delta }}}^{2}\omega \rangle }_{t| k}+{\tilde{\sigma }}^{2},$$

where the two ODEs for the mean and the variance now replace the SDE, Eq. 3, and *t*_*k*_ ⩽ *t* ⩽ *t*_*k*+1_, i.e. the evolution in time is between two successive measurements at *t*_*k*_ and *t*_*k*+1_. As said, the conditioning index *k* refers to the fact that the update with data at *t*_*k*_ moves the evolution to new initial points, e.g. *ω*_*B*_ → 〈*ω*〉_*k*∣*k*_, Fig. 1b. When *k* = 0, there are no updates with measurements (Table S2), and the ODEs evolve from the initial values at *t* = 0; specifically, Eq. 18a with *B* = 0 then becomes the usual ODE for the mean value of the cake, known in membrane science.

In literature, Eqs. 18a and 18b are usually given in the most general matrix format needed to handle multiple random states, e.g., Ref. ^29^ (p.26). Our case of a single (scalar) hidden state allows for insightful reduction. Note that our notation 〈*x*〉 replaces literature symbols $\hat{x}$. In the steady-state the above equations yield 〈*ω*〉_*ss*_ = *Bu*_*t*_/( − *A*) and ${\langle {{{\Delta }}}^{2}\omega \rangle }_{ss}={\tilde{\sigma }}^{2}/(-2A)$, respectively. The first is the basis for the input-driven control, and the second is the attainment of the finite variance (finite uncertainty spread) in the long term.

The output prediction equations are (from Eqs. 1 and 17b, and S16)

19a $${\langle J\rangle }_{k| k-1}=C{\langle \omega \rangle }_{k| k-1}+D{u}_{k},$$

19b $${\langle {{{\Delta }}}^{2}J\rangle }_{k| k-1}={C}^{2}\left\langle \right.{{{\Delta }}}^{2}\omega {\rangle }_{k| k-1}+{S}_{k}.$$

The one-step ahead prediction of the flux into the current step, 〈*J*〉_*k*∣*k*−1_, is a linear combination of the mean of the state 〈*ω*〉_*k*∣*k*−1_ from the previous step and the input *u*_*k*_ from the current step. Similarly, the one-step ahead prediction of the variance ${\langle {{{\Delta }}}^{2}J\rangle }_{k| k-1}$ is a linear combination of the variance of the state ${\langle {{{\Delta }}}^{2}\omega \rangle }_{k| k-1}$ from the previous step (model uncertainty or the process noise) and the measurement-error variance *S*_*k*_ of the current step (the measurement noise).

Since in the current step *t*_*k*_ the measurement ${{{{{{{{\mathcal{J}}}}}}}}}_{k}$ generally differs from the predicted 〈*J*〉_*k*∣*k*−1_, Fig. 1b, the update of the latter with the former is done to get 〈*J*〉_*k*∣*k*_. This is effected by updating the hidden state, our primary variable, from 〈*ω*〉_*k*∣*k*−1_ to 〈*ω*〉_*k*∣*k*_. Linear interpolation gives the update equations

20a $${\langle \omega \rangle }_{k| k}={\langle \omega \rangle }_{k| k-1}+{K}_{k}\left({{{{{{{{\mathcal{J}}}}}}}}}_{k}-{\langle J\rangle }_{k| k-1}\right),$$

20b $${\langle {{{\Delta }}}^{2}\omega \rangle }_{k| k}=(1-{K}_{k}C){\langle {{{\Delta }}}^{2}\omega \rangle }_{k| k-1},$$

where the factor *K*_*k*_ is the point-dependent Kalman gain

20c $${K}_{k}=\frac{C{\langle {{{\Delta }}}^{2}\omega \rangle }_{k| k-1}}{{C}^{2}{\langle {{{\Delta }}}^{2}\omega \rangle }_{k| k-1}+{S}_{k}}=\frac{1}{C+\frac{{S}_{k}}{C{\langle {{{\Delta }}}^{2}\omega \rangle }_{k| k-1}}}.$$

To get Eq. 20b and c, we put Eqs. 1 and 19a into Eq. 20a and minimize the obtained variance ${\langle {{{\Delta }}}^{2}\omega \rangle }_{k| k}$, see Variances, Supplementary Note 11.

If the measurement error is zero, *S*_*k*_ = 0, the measurements become absolutely precise; in that case *K*_*k*_ = 1/*C*, ${\langle {{{\Delta }}}^{2}\omega \rangle }_{k| k}=0$, ${\langle \omega \rangle }_{k| k}={{{{{{{{\mathcal{J}}}}}}}}}_{k}/C$ and ${\langle J\rangle }_{k| k}={{{{{{{{\mathcal{J}}}}}}}}}_{k}$, i.e. the updated state and observable are made up of the measurement value, the model being irrelevant for the update. On the other hand, if ${\langle {{{\Delta }}}^{2}\omega \rangle }_{k| k-1}=0$ (the uncertainty in the model is zero i.e. a deterministic ODE, not an SDE, describes the state), the system’s model becomes absolutely precise; then, *K*_*k*_ = 0, ${\langle {{{\Delta }}}^{2}\omega \rangle }_{k| k}={\langle {{{\Delta }}}^{2}\omega \rangle }_{k| k-1}$, 〈*ω*〉_*k*∣*k*_ = 〈*ω*〉_*k*∣*k*−1_ and 〈*J*〉_*k*∣*k*_ = 〈*J*〉_*k*∣*k*−1_, i.e. the updated state and observable are those of the pure model and the measurements are disregarded in the update. Here the values are independent of _∣*k*_, hence 〈*ω*〉_*k*∣0_ = 〈*ω*〉_*k*∣*k*−1_ and 〈*J*〉_*k*∣0_ = 〈*J*〉_*k*∣*k*−1_ i.e. the long-term predictions coincide with the short-term predictions in the ODE case. Thus, deterministic ODE models are a special case of the more general SDE approach. In reality, 〈*ω*〉_*k*∣*k*_ is in between the two bounding values, ${{{{{{{{\mathcal{J}}}}}}}}}_{k}/C$ and 〈*ω*〉_*k*∣*k*−1_ (${g}^{-1}({{{{{{{{\mathcal{J}}}}}}}}}_{k})$ and *ω*_*B*_ in Fig. 1c, respectively).

All what is said is valid exactly for the linear systems. When *f* and *g* are non-linear (as in our case of ultrafiltration), non-Gaussian distributions arise, and the filtering is no longer exact since 〈 *f*(*x*)〉 ≠ *f*(〈*x*〉) in non-linear case. We Taylor-expand the equations around the Gaussian mean to use the formalism. This is known as the Extended Kalman Filter (EKF)^26^. All the equalities are now only approximately true. For example Eqs. 18a,b become

21a $$\frac{d{\langle \omega \rangle }_{t| k}}{dt}\approx f\left({\langle \omega \rangle }_{t| k}\ldots \right),\quad {t}_{k} \; \leqslant \; t \; \leqslant \; {t}_{k+1}$$

21b $$\frac{d\left\langle \right.{{{\Delta }}}^{2}\omega {\rangle }_{t| k}}{dt}\approx 2{A}_{t}\left\langle \right.{{{\Delta }}}^{2}\omega {\rangle }_{t| k}+{\tilde{{\sigma }_{t}}}^{2},\quad {t}_{k} \; \leqslant \; t \; \leqslant \; {t}_{k+1}$$

where ${A}_{t}=(\partial f/\partial \omega ){| }_{\omega = {\langle \omega \rangle }_{t| k-1}}$. Similarly, the coefficient *C* becomes ${C}_{k}=(\partial g/\partial \omega ){| }_{{\omega }_{k} = {\langle \omega \rangle }_{k| k-1}}$.

Note that the non-linear character of *f* is preserved; it is the equation of variance of the state (via the coefficient *A*), and the equations of mean and variance of the flux (via the coefficient *C*) which are modified. The formal account can be found in^29^(p.28).

The entire procedure of the Kalman filtering—the state predictions, the output predictions and the updates with the Kalman gain—lowers the dispersion of random processes as predictions are updated with data. In doing so, the method apportions the optimal weights between the measurement error and the model-related uncertainty. In other words, if the initial conditions are Gaussian and the processes linear, the filtered state and output predictions remain Gaussian; if further the measurement errors are Gaussian, the filtered updates are Gaussian, too. In such case it is possible to optimally divide the uncertainty between the measurement error *S*_*k*_ and the process noise ${\langle {{{\Delta }}}^{2}\omega \rangle }_{k| k-1}$ to yield the minimal variance of the updated state. The filtering ensures the optimal ‘positioning’ of the modelled state and is essential in control theory where one must constantly correct predictions with data updates (section Control).

The variance of the updated hidden state is minimized under the Kalman gain, Eq. 20c. The optimal gain is function of the *θ* parameters *S*_*k*_, *A* etc. Those parameter values which in addition obtain the maximum of the likelihood are chosen as the best model parameters (likelihood is not part of the filtering process, see next section). Different models, with different values of their best parameters, will yield different Kalman gains, and thus different corrections in data updates.

The (extended) Kalman filtering is part of many data-based predictive statistical algorithms, e.g.^42^, and is fully implemented in our user-friendly software CTSM-R (Continuous Time Stochastic Modelling for R)^27,29,43^.

### Likelihood and statistical validation

The term in the parentheses of Eq. 20a is called innovation error (or the one-step ahead residual)

22 $${\epsilon }_{k}={{{{{{{{\mathcal{J}}}}}}}}}_{k}-{\langle J\rangle }_{k| k-1},$$

as it quantifies the difference between the measured and the one-step ahead predicted value of observable *J* in step *k*. The likelihood function is the product of the Gaussian weighted distributions of the innovation errors

23 $$L({{{{{{{\boldsymbol{\theta }}}}}}}};{{{{{{{{\mathcal{J}}}}}}}}}_{N})=\mathop{\prod }\limits_{k=1}^{N}\frac{\exp \big(-\frac{1}{2{\langle {{{\Delta }}}^{2}J\rangle }_{k| k-1}}{\epsilon }_{k}^{2}\big)}{\sqrt{2\pi \,\det ({\langle {{{\Delta }}}^{2}J\rangle }_{k| k-1})}}.$$

In our single-state case ${\langle {{{\Delta }}}^{2}J\rangle }_{k| k-1}=\langle {\epsilon }_{k}^{2}\rangle$, Eq. S16. Using the logarithm we obtain

24 $$\ln L({{{{{{{\boldsymbol{\theta }}}}}}}};{{{{{{{{\mathcal{J}}}}}}}}}_{N})=-\frac{1}{2}\mathop{\sum }\limits_{k=1}^{N}\left(\frac{{\epsilon }_{k}^{2}}{\langle {\epsilon }_{k}^{2}\rangle }+\ln \langle {\epsilon }_{k}^{2}\rangle +\ln 2\pi \right),$$

where *ϵ*_*k*_ = *ϵ*_*k*_(*θ*), i.e. the error is a function of parameters. ${\epsilon }_{k}^{2}(\theta )$ are the quadratic residuals that depend on the non-random terms of the models (Eqs. 22 and 19a), while $\langle {\epsilon }_{k}^{2}(\theta )\rangle$ is the variance measuring the model- and the measurement uncertainties (Eq. 19b).

The optimal parameters maximize the log likelihood (*ll*) (minimize the terms in parentheses of Eq. 24) and are found numerically

25 $$\hat{{{{{{{{\boldsymbol{\theta }}}}}}}}}=\arg \mathop{\max }\limits_{\theta \in {{\Theta }}}\{\ln \left(L({{{{{{{\boldsymbol{\theta }}}}}}}};{{{{{{{{\mathcal{J}}}}}}}}}_{N})\right.\}.$$

Different models yield different maxima of the likelihood and hence different parameters. Thus, parameters reflect differences in models, quantified by the likelihood scores. Modelling of the uncertainty, e.g. Eq. 6a, makes it possible to assign larger variances to large residuals i.e. weight less the larger errors making the first term in Eq. 24 smaller, thus increasing the likelihood; otherwise, all errors are weighted equally (ODE cases), and likelihood decreases.

The likelihood pertains to the short-term predictions, Eq. 22, which are computationally cheap, and is thus one of the main quantitative measures (guidelines) in statistics for model comparison (larger the (log)likelihood, better the model).

Statistical validation is the statistical comparison of model predictions against all measurements, in our case the 23 time-series of Figs. 1a and S4a. We use two statistical measures for that purpose: the likelihood based Akaike Information Criterion (AIC), and the root-mean-square error (RMSE).

The Akaike Information Criterion is given by

26 $$AIC=2k-2{{{{{{{\rm{\ln }}}}}}}}\left(L({{{{{{{\boldsymbol{\theta }}}}}}}};{{{{{{{{\mathcal{J}}}}}}}}}_{N})\right.,$$

where *k* is the number of parameters, or degrees of freedom (*df*). A model with larger number of parameters producing the same log likelihood is poorer. For our systems with a few parameters (*df* ~ 10), AIC is essentially twice the negative *ll*. Hence, the smaller the AIC, the better the model.

The root-mean-square error is given by

27 $$RMSE={\left[\frac{1}{N}\mathop{\sum}\limits_{i}\mathop{\sum}\limits_{k}{({{{{{{{{\mathcal{J}}}}}}}}}_{i,k}-{\langle J\rangle }_{i,k| 0})}^{2}\right]}^{1/2},$$

where *i* goes over all time-series (data sets), and *k* over time. *N* is the total number of data points. RMSE is the ordinary least-square measure of the goodness of a fit for an ODE, and compares how much the measurements deviate from the long-term predicted mean.

In the case of ODEs 〈*J*〉_*k*∣*k*−1_ = 〈*J*〉_*k*∣0_, and so maximizing the *ll* and minimizing the RMSE becomes one and the same condition (Eqs. 22, 24, and 27). For SDEs the two are different. Whereas the *ll* scores reflect parameters, the RMSE scores reflect the structure of the model equations and are used here to asses the models’ long-term predictions. The validation of the models in this article is shown in Tables 1 and S3 for the real and the illustrative models, respectively.

### The Lamperti transform

With transform

28 $${z}_{t}=\log ({\omega }_{t})\Rightarrow {\omega }_{t}={e}^{{z}_{t}},$$

Eq. 5 becomes

29 $$d{z}_{t}={c}_{b}{e}^{-{z}_{t}}({J}_{t}-{J}_{ss}(1-{e}^{-{e}^{{z}_{t}}/{\omega }_{c}}))dt$$

30 $$-\frac{1}{2}{\sigma }^{2}(t)dt+\sigma (t)d{W}_{t},$$

and the state-dependence of the diffusion is removed. The integration then produces the log-normal distribution of the state.

### Expansion of *P*_*t*_ and *Q*_*t*_ in a basis set

We expand Δ*P*_*t*_ and *Q*_*t*_ into Legendre polynomials and then find the coefficients of the expansion which satisfy the constraint Eq. 13b. For example, the polynomials representing *Q*_*t*_ are (for *t*_0_ ⩽ *t* ⩽ *T*)

31 $${P}_{Q}(t)={a}_{0}{L}_{0}(\tilde{t})+{a}_{1}{L}_{1}(\tilde{t})+\ldots {a}_{p}{L}_{p}(\tilde{t}),$$

where *a*_*i*_ are coefficients of the Legendre polynomials ${L}_{i}(\tilde{t})$ of *i*-th order, and

32 $$\tilde{t}=\frac{2[t-\frac{1}{2}(T+{t}_{0})]}{T-{t}_{0}}$$

is the time scaled to the orthogonality interval [−1, 1]. Legendre polynomials up to the 4th order are used.

We further restrict the range of our control variables Δ*P*_*t*_, *Q*_*t*_ by the following scaling transformation

33a $${Q}_{t}=\frac{{R}_{Q}}{1+{e}^{-{P}_{Q}(t)}}+{Q}_{min},$$

33b $${{\Delta }}{P}_{t}=\frac{{R}_{{{\Delta }}P}}{1+{e}^{-{P}_{P}(t)}}+{{\Delta }}{P}_{min},$$

which limits the values to *Q*_*min*_ ⩽ *Q*_*t*_ ⩽ *Q*_*min*_ + *R*_*Q*_ and Δ*P*_*min*_ ⩽ *P*_*t*_ ⩽ *P*_*min*_ + *R*_Δ*P*_.

### Supplementary information

Goranovic_PR file

Supplementary Information

## Data Availability

The data that support the findings of this study are subject to contractual restrictions signed between Grundfos Holding A/S and Technical University of Denmark. Data will be made available by the authors upon reasonable request and with received permission from Grundfos Holding A/S.

## Code availability

The R code used for modelling of data is at 10.6084/m9.figshare.20231451.v1.
