# Supplementary material for: A data-driven digital twin for water ultrafiltration
Source: Commun Eng. 2022 Sep 30;1:23. doi: 10.1038/s44172-022-00023-6 (PMC10956058; doi:10.1038/s44172-022-00023-6)
Supplement: Supplementary file 2 — Supplementary Information [file 44172_2022_23_MOESM2_ESM.pdf]

A data-driven digital twin for water ultrafiltration: Supplementary file

Goran Goranović[a,*], Jan Kloppenborg Møller[a,*], Per Brath[b,**], Henrik Madsen[a]

[a]DTU Compute, Asmussens Allé Building 303B, Kgs. Lyngby DK-2800
[b]Grundfos A/S, Poul Due Jensens Vej, Bjerringbro DK-8850

Abstract

The supplementary file contains details of experiments as well as additional modeling and control material. Supplementary references are separate from those in the main file, and are given at the end of the supplement. The same references can thus appear under different numbers in the two documents.

S1. Supplementary Method 1

S1.1. Supplementary Note 1

Our experimental set-up is schematically shown in Fig. S1b.

We used inopor® ultra flat-sheet TiO_2 ceramic membrane for ultra-filtration with pore size of 10 nm and dimensions $(L_\mathrm{m}, W_\mathrm{m}, H_\mathrm{m})$= (10 cm x 5 cm x 2 mm). The membrane was mounted inside the chamber of inner dimensions $(L_\mathrm{c}, W_\mathrm{c}, H_\mathrm{c})$=(9.5 cm x 4.5 cm x 4 cm) with active layer facing the flow. The membrane's active area was thus $A \simeq 43$ cm^2.

An inlet and an outgoing pipe 3.8 cm in diameter brought the liquid in contact with the membrane. Maximum Reynolds number (velocity corresponding to $Q = 3.5$ L/h going through the inlet pipe), based on chamber's hydraulic diameter $D_h = 2L_c \cdot W_c/(L_c + W_c) = 6.1$ cm, was $Re_\mathrm{max} = 52$, hence we had laminar flow.

Pressure, P, and cross-flow, Q, were regulated by two separate pumps actuated by computer, and measured by the indicated sensors. Stability of the input sequences of the variable ΔP and Q (input data) was secured by PI controllers programmed in MATLAB and Python. The permeate flux (output data) was determined by weight. The system had a heat exchanger installed to regulate the temperature of the water, $T = (22.4 \pm 0.5)$ °C.

The membrane's native resistance, R_m, was determined on clean water experiments on a batch of 14 membranes. $R_m = (0.59 \pm 0.04)$ [bar h L^{-1}], (or in SI units, $R_m = (9.1 \pm 0.6) \cdot 10^{11}$ [m^{-1}], Sec. S3.2, Tables S4 & S5). The permeability per unit area is thus $A = (40 \pm 3) \cdot 10$ [L h^{-1} bar^{-1} m^{-2}] at $T = (22.4 \pm 0.5)$ °C.

As shown in Fig. S1b, there is a circulation of the retentate back to the reservoir. However, the bulk concentration changed a little over the course of a single measurement, as three liters (six at most) out of 77 were typically filtered in a measurement; the change in volume was thus 4-8%, but the mass of the lakewater dirt was also lowered by the amount collected in the surface filtrate. Concentrations of various ingredients are given in the recipe of Table S1 below.

S1.2. Supplementary Note 2

We mimicked dirty lake water by mixing proteins (BSA), polysaccharides (sodium alginate, S.-Aldrich; MW = 12kDa - 40kDa), minerals (various salts and kaolinite), and soil (humic acid, S.-Aldrich; consisting of heteropolycondensates of MW = 2 kDa - 500 kDA, but mainly 20 - 50 kDa) in ultra pure water (milli-Q), see Table S1.

The mixture is very complicated and generally inhomogeneous, so the number given in Table S1

*These authors contributed equally; gogo@dtu.dk, jkmo@dtu.dk

**Present address: Danfoss Drives A/S, Global R&D, Design Center Denmark, Aalborg Ø, DK-9220

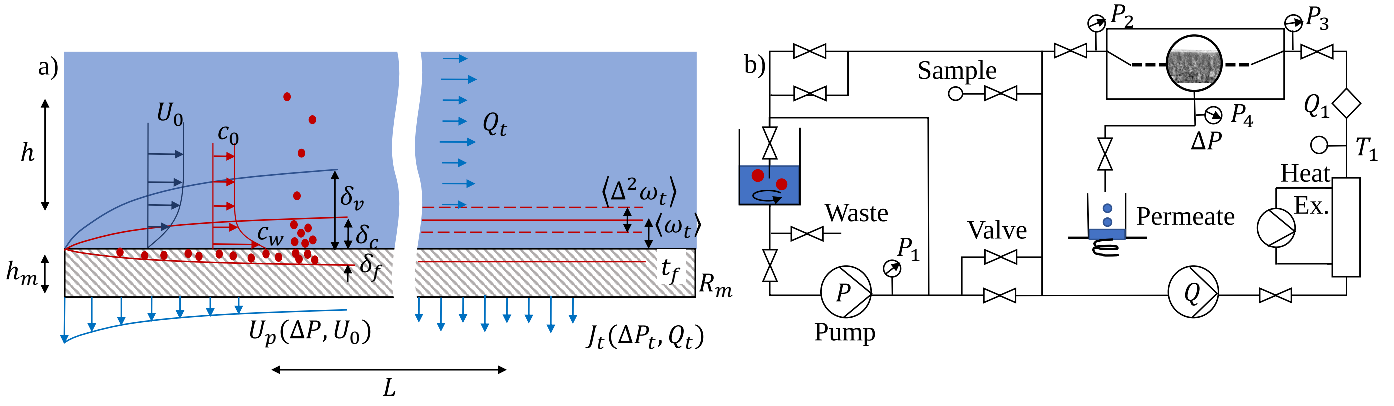

Figure S1: Cross-flow ultrafiltration. a) Traditional boundary-layer approach to cross-flow ultrafiltration (left) and the statistical approach of this study (right). The statistical approach is not spatially resolved but enables handling of complicated time dependent inputs/outputs and the explicit account of uncertainties (Symbols: U_0, U_p - free-stream cross-flow and permeate velocity, respectively, c_0, c_w - bulk and wall concentrations, δ_v, δ_c, δ_f - velocity-, concentration-, and fouling-boundary layers, h, L - chamber height and length, h_m - membrane thickness; Q_t, ΔP_t - time-dependent cross-flow and pressure, respectively, J_t - permeate flux, $\langle \omega_t \rangle$ - average filtrate thickness, $\langle \Delta^2 \omega_t \rangle$ - filtrate thickness variance, t_f - time scale of fouling, R_m - membrane resistance). b) Schematic of the set-up, featuring two separate pumps to control the pressure and cross-flow across a rectangular ultrafiltration membrane (inset). The system had a heat exchanger installed to regulate temperature of water. Transmembrane pressure is $\Delta P_{TMP} = (P_2 + P_3)/2 - P_4$. Q_1 and T_1 are the cross-flow and temperature sensor, respectively.

Table S1: Recipe of the artificially simulated lakewater. MW - molecular weight

Material	MW [kDa]	m [g]	V_{water} [L]	c [g L^{-1}]
Protein/ polysaccharides				
BSA	66.5	4.7		$6.1 \cdot 10^{-2}$
Na^+Alginate	12-40	3.8		$5.0 \cdot 10^{-2}$
in milliQ H_2O			0.2	
Humic acid	2-500	0.81		$1.1 \cdot 10^{-2}$
in milliQ			1	
Salts/clay				
$NaHCO_3$		4.3		$5.6 \cdot 10^{-2}$
NaCl		9.3		$1.2 \cdot 10^{-1}$
Na_2SO_4		3.0		$3.9 \cdot 10^{-2}$
$CaCl_2$		0.056		$7.2 \cdot 10^{-4}$
kaolinite		0.0083		$1.1 \cdot 10^{-4}$
in milliQ			0.5	
all added to demin. water			75	
total		26	76.7	$3.4 \cdot 10^{-1}$

for total bulk concentration (total mass over total volume), $c_b = 0.34$ g L^{-1}, is for orientation only, to give us the feel for the magnitude of SI parameters in Table S5, namely for α_0 which is unknown for our system and few other that depend on it (the uncertainty in concentrations c_b obtained from models is $\sim 13\%$, Table 1). We can then easier compare our values with literature, end of Sec. S3.3. Note that our modeling does not require detailed knowledge of concentration beforehand. In real-world control scenarios, where conditions are much less controlled than in our experiments here, changes in parameters are accommodated by new parameter estimations which are re-done regularly, say, every other day, from which a new concentration can be determined. This is how a waste-water control has been effected in a plant in Denmark, [1].

We observed at the end of each measurement that a (thin) brownish tar-like gel formed at the membrane's surface, forcing us to chemically clean the membranes after each run. The consistency of the gel was similar to the stain of evaporated coffee. The color stems likely from all three ingredients - the kaolinite, the alginate and the humic acid - with last one probably contributing the darkest hue.

The decisive ingredient for the control of gelation was $CaCl_2$, on two accounts: 1) as a known desiccant it bounds water in the form of various hydrates e.g. $CaCl_2 \cdot 6H_2O$, and 2) Ca^{2+} ions replace sodium to cause cross-linking of different al-

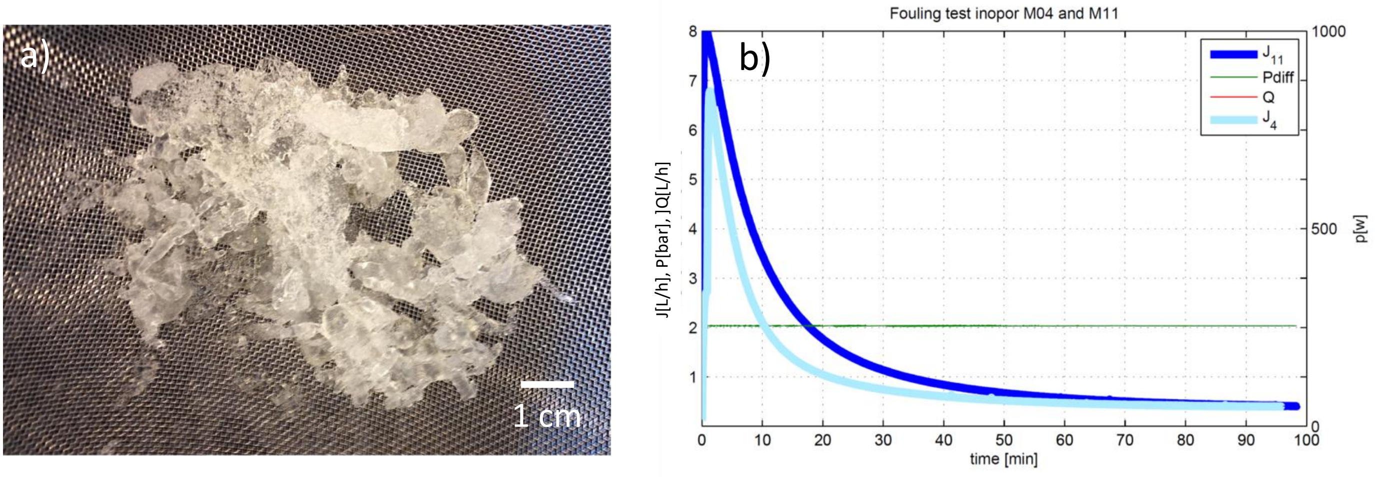

Figure S2: Experimental evidence of fouling. a) Gel formation mediated by $CaCl_2$ b) Smoothed permeate fluxes through two different membranes at constant ΔP and Q inputs; an experimental test for adjustment of c($CaCl_2$). The mass balance is practically achieved by 100 min. These types of measurements were not used in our analysis as they were unsuitable (not having enough variability) for real-time control purposes of Sec. 2.7.

ginate chains. Type of watery gel formed is shown in Fig. S2a. We had to do preliminary filtration experiments to adjust (minimize) the amount of $CaCl_2$, and hence the amount of fouling, since too much of it caused gelation throughout the entire piping system. These experiments were with constant input e.g. shown in Fig. S2b. Few modeling results for constant input values are in Fig. S3.

We have not analyzed the permeate (not the focus of the study), but based on the inopor® product data-sheet the cut-off for the ultrafiltration membrane with 10 nm pores is 20 kDa (under 3-8 bar). A very high percentage of the material was thus retained, especially if one considers the predominant polymer entanglement mediated by $CaCl_2$.

From the above discussion we can argue that our cake consisted of irreversible hard polymer mesh imbued with $CaCl_2$ hydrates and other salts which caused osmotic pressure and reversible swell and compression of the mesh. In other words, the last two ingredients likely responded instantaneously to abrupt changes in pressure and cross-flow, making the oscillations in the flux and the cake's thickness visible from data and model predictions, Fig. 2 (the variations reflect also variability of different membranes, of course). However, the irreversible component was also there.

Thus, both the cake and the concentration polarization contributed to lowering of the permeate flux. Each of the two phenomena are indeed at play in ultrafiltration [2]. Note that our modeling results remain unaffected by the nature of the filtrate since the modified Darcy's resistance approach that we use in our models implicitly accounts for both mechanisms (not distinguishing between the two), as shown e.g. in [3].

S1.3. Supplementary Note 3

The 20 out of 23 randomized sequences of time-dependent pressure and cross-flow inputs are shown in Fig. S4a.

The two beta distributions for randomizing the time between shifts in the series are shown in Fig. S4b: gamma distribution with parameter $\alpha = 6$ and $\beta = \frac{6}{230}$ (black line; series 1-10), and parameters $\alpha = 12$ and $\beta = \frac{12}{460}$ (red line, series 11-20). See Methods, Sec. 4.1.

S2. Supplementary Method 2

Stochastic greybox modeling combines noisy data with stochastic physical processes, [4–6], in contrast to purely data-driven statistical blackbox modeling (e.g. ARMAX - AutoRegressive Moving

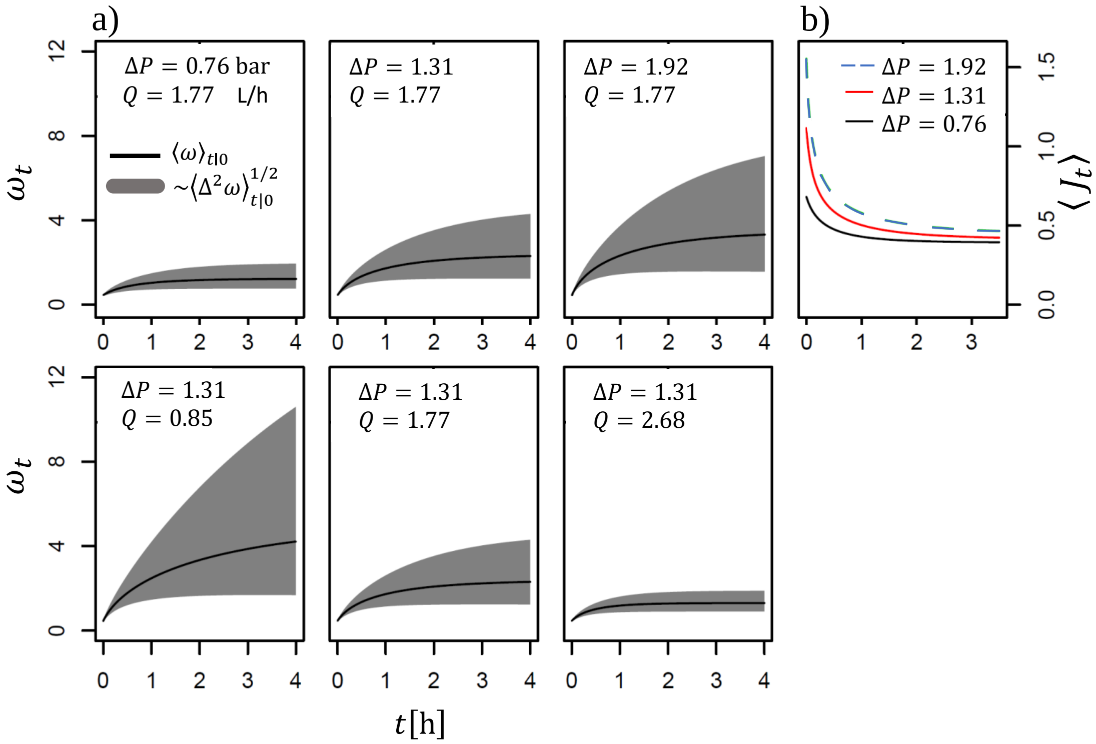

Figure S3: Model $M6$ predictions for constant inputs. a) States (cakes) for a series of constant ΔPs and a fixed Q (top row), and for a series of constant Qs and a fixed ΔP. The states in the top leftmost and bottom rightmost are similar, but differ in consumed energy. Large prediction intervals (grey areas) include also the variability of different membranes. b) Fluxes corresponding to the top row.

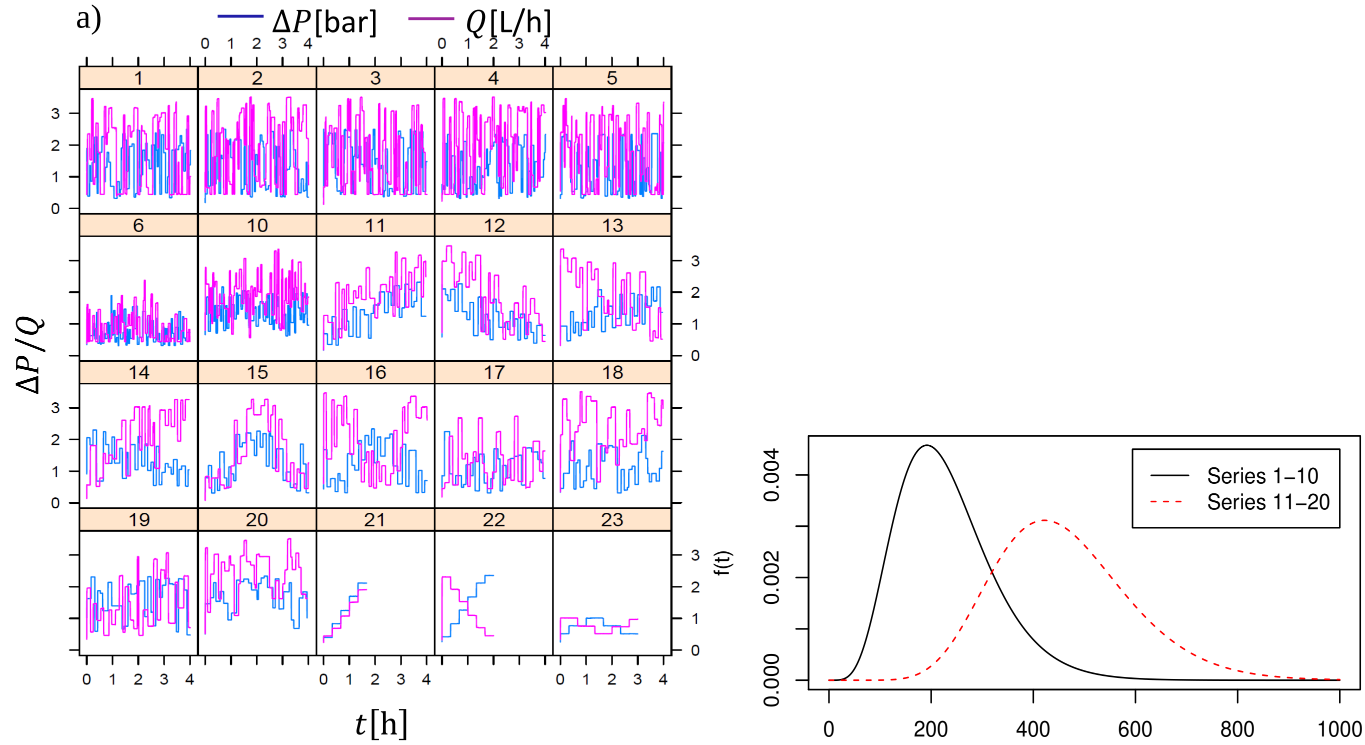

Figure S4: Randomization. a) Input data series of pressure ΔP and cross-flow Q have been designed (programmed) to provide diverse scenarios for statistical analysis. The pressure ranged between $(0.5, 2.6)$ bar, and the cross-flow between $(0.5, 3.5)$ [L h^{-1}]. The series $1-10$, $11-20$ and $21-23$ are distinguished by temporal changes in the range of $1/10$, $1/4$ and $1/2$ of an hour, respectively. b) Distributions to model the time between shifts for the different series, see Methods Sec. 4.1.

Average with eXogeneous input), or purely deterministic physical whitebox modeling (the usual physical theory and phenomenology e.g. ODEs, Ohm's law etc.). The modeling deals with two types of variables: directly observable variables (in our case flux through a membrane), which are measured as discrete time-series, and unobservable ones (usually not observed within given set of experiments e.g. thickness of the filtrate at the membrane), also known as hidden states, which run in the background as continuous-time random (Wiener) processes. In general, there are multiple hidden states to a problem. The hidden states evolve by SDEs, whereas the measured observables are algebraic functions of the integrated hidden states. Both the differential equations as well as the algebraic functions are subject to modeling and are in general non-linear.

Two types of uncertainties are present: measurement errors and hidden state diffusion. The diffusion is not only a physical diffusion but also quantifies the unknowns about the system's state. The task of the modeling is to properly apportion and then to quantify the two uncertainties from data. This is done using the method of Kalman filtering [7–9], which enables that the model predictions are optimally updated with data and then used in real-time control scenarios. The model parameters are found using the maximum likelihood estimate, a generalized least square method [9].

The modeling provides equations not only for the mean values of physical variables but for their covariances as well (as in the stochastic damped harmonic oscillator, [10]). In the greybox modeling the exact model may be unknown, hence candidate equations are proposed and statistically evaluated. It is similar to ODE modeling of chemical kinetics, where both the (unknown) equations of reaction mechanisms and the (unknown) rate constants/parameters appearing in them need to be determined to best fit given data sets. The advantage of the greybox models is the explicit account of model uncertainties, covariances, implementation of control via optimal updates, and often a smaller number of parameters.

S2.1. Supplementary Note 4

In a time series, the values of ω at time t_k, is denoted as ω_k, where the discrete index right next to a variable is used when the measurements are equidistant in time (the most common case). ω_k can be predicted (by integrating the state model equation) taking none, some, or all measurements into account. We write $\omega_{k|0}$, $\omega_{k|k-l}$, $\omega_{k|k}$ (pronounced 'ω_k given $k-l$', 'ω_k given k' (measurements) etc.). These represent the k-step ahead, the l-step ahead and the zero-step ahead prediction, respectively. The last is also called 'update'. This is summarized in Table S2.

Table S2: Nomenclature explanation. The hidden state ω and the observable J (t is continuous, k discrete).

Symbol	Reading (Meaning)
$\omega_{t\|k}$	ω at t, given measurements (of $\mathcal{J}$) up to t_k (conditioning on measurements up to t_k)
$\omega_{t\|0}$	ω at t, given no measurements (t-step ahead, or long-term prediction)
$\omega_{k\|k-1}$	ω at t_k, given measurements (of $\mathcal{J}$) up to t_{k-1} (one-step ahead, or short-term prediction)
$\omega_{k\|k}$	ω at t_k, given measurements (of $\mathcal{J}$) up to t_k (the update with the current-step data)
$J_{k\|k-1}$	J at t_k, given measurements of $\mathcal{J}$ up to t_{k-1} (one-step ahead prediction of the observable)

The short-term predictions (the one-step ahead predictions, i.e. $\omega_{k|k-1}$, $J_{k|k-1}$), are easy to compute and are thus used for the likelihood and optimal parameters (Methods, Sec. 4.3); however, there is no guarantee that the long term predictions of a model using these parameters will correctly guess the long term data trends (divergence can and does occur in the long term due to misspecification of model structure). Hence, modeling must correctly fix the long-term trends. We are thus interested in both $\omega_{k|k-1}$ and $\omega_{k|0}$, for parameter estimation and testing of the model structure (used in predictive control later on), respectively.

Said differently, the state (cake) is estimated only by the state model, in our case Eq. 5, but its predictions are then adjusted by conditioning them on (flux) data: predictions labeled $_{|0}$ differ from those labeled $_{|k}$, Table S2. The conditioning

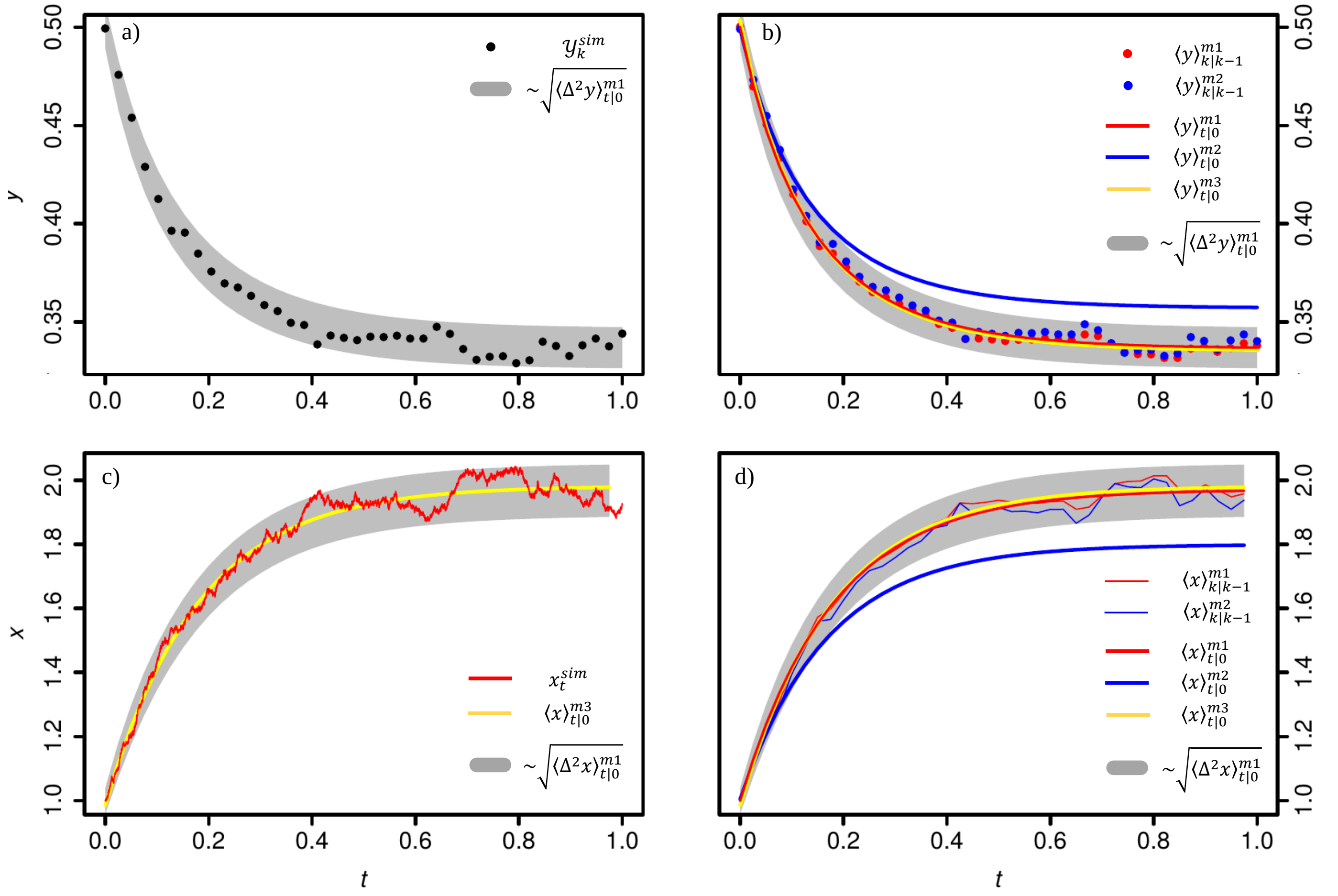

Figure S5: An example with 40 simulated observations. Superscripts refer to models. a) The observed flux y (dots) and its prediction interval (95.4 % or $\pm 2\sqrt{\langle \Delta^2 y \rangle_{t|0}}$, grey area). b) The one-step ahead predictions of the flux, used for parameters (dots), and the corresponding long-term predictions (lines); note that in ODE case (yellow), dots and lines coincide $y_{k|0} = y_{k|k-1}$. c) A stochastic realization of the cake x (red) leading to the flux in panel a), and the cake's prediction interval (grey). ODE of $m3$ provides only the mean (yellow), without uncertainty. d) The one step ahead predictions (thin lines) and the long-term predictions of the cakes (thick lines). In state-based modeling, observables always go via states: c) yields a) and d) yields b).

is important because it is the update of the virtual states of the digital twin by data.

S2.2. Supplementary Note 5

Consider the following hidden state and observation model equations

$$dx_t = A(\mu - x_t)dt + \sigma dW_t, \qquad \text{(S1)}$$

$$y_k = \frac{1}{1 + x_k} + e_k, \quad e_k \sim N(0, S) \qquad \text{(S2)}$$

where A, μ, σ, the initial state x_0 and the variance S of the measurement error are parameters. Eq. S1 is the exponential stochastic reversion of the state x to the mean μ[1], and Eq. S2 a non-linear observation equation (equivalent to the Darcy's law for the flux y at constant pressure of 1).

The data in Figs. S5a and 5c is in fact generated using the above equations. With $(A, \mu, \sigma, x_0)_0 = (5,\ 2,\ 0.2,\ 1)$, we used Eq. S1 to generate a stochastic realization, jagged red line in c), and then used Eq. S2, with S=0.001, to generate 40 observation points in a). In other words, Eq. S1 is the true model of the hidden state.

We now apply CTSM-R [12] to fit and test three models to the 40 'measured' datapoints. All proposed models are variants of Eq. S1: $m1$ is the actual true model with five unknown parameters $(A, \mu, \sigma, x_0, S)_1$, and its fit recovers the above true parameters. The mean values and the pre-

[1]Known as the Ornstein-Uhlenbeck (O-U) process, [11].

diction intervals of $m1$ are shown in red and grey in Fig. S5; $m2$ assumes a different steady-state mean, $\mu = 1.8$, and has four unknown parameters $(A, \sigma, x_0, S)_2$ (in blue); $m3$ is an ODE model, i.e. $\sigma = 0$, and has also four parameters, $(A, \mu, x_0, S)_3$ (yellow). Table S3 lists their statistical scores.

Table S3: Validation of models in the example. The log-likelihood (ll) and the Akaike Information Criterion (AIC) evaluates the short-term prediction, and the root-mean-square error (RMSE) the long-term prediction. *df* - degrees of freedom i.e. number of parameters. Details in Sec. 4.3.

	ll	df	AIC	RMSE
$m1$	164.9	5	-319.8	0.0043
$m2$	155.4	4	-302.8	0.019
$m3$	162.9	4	-317.9	0.0041

From the table we see that $m1$ has the largest log-likelihood (ll) and hence the most correct parameters (See Eq. 25 and the related discussion, Methods Sec. 4.3), as it should be for the true model. Model $m3$ has somewhat less accurate parameters, i.e. its ll is smaller compared to $m1$ (the error $S_3 = 0.004$ comes out four times larger than the true value to compensate for $\sigma = 0$, and likelihood is reduced). Note that $m3$ has the smallest root-mean-square error (RMSE).

As seen from Fig. S5, the long-term means of $m1$ (thick red line) and $m3$ (thick yellow line) models are almost identical, panels b) and d), but $m3$ does not account for cake's uncertainty, panel c). The cake's uncertainty (prediction interval) of model $m1$ (95.4%, $\pm 2\sqrt{\langle \Delta^2 x \rangle_{t|0}^{m1}}$, in grey) grows until reaching the finite extent in the steady state ($\pm 2\sqrt{\sigma_1^2/2A_1}$).

Model $m2$ has a fixed $\mu_2 = 1.8$, significantly off the data trend, illustrating that a model's long-term prediction of the mean will generally fit some series worse than others (if fitting multiple series). This is of course reflected in the scores.

Finally, with real observables one is never certain of the true state model – hence the greybox approach. In the example, the closest one gets to the behavior of the cake are the one-step ahead predictions $\langle x \rangle_{k|k-1}$ of a particular model, thin lines panel d), which take into account the $k-1$ previous measurements of the flux. Note the similarity between $\langle x \rangle_{k|k-1}$ and the Monte Carlo realization that is unavailable with real data. From $\langle x \rangle_{k|k-1}$ one obtains $\langle y \rangle_{k|k-1}$ and uses these to maximize the likelihood and calculate the parameters, Methods Sec. 4.3.

For the Example's script, see the Code Availability statement.

S3. Supplementary Discussion

S3.1. Supplementary Note 6

Our equations are templated on the study [13] (and references therein). The permeate flux there is the usual Darcy equation

$$J = \frac{\Delta P}{\mu(R_0 + R_c)}, \tag{S3}$$

with μ dynamic viscosity, and R_0 and R_c the membrane and the cake resistances, all measured in SI units. Comparing Eq. S3 with our Eq. 2, we note that in our flux model μ is incorporated into Rs. From [13], the above R_c is given as

$$R_c = \alpha\omega, \tag{S4}$$

where α is the specific resistance to filtration, and ω the filter cake mass per area (our hidden state). The specific resistance linearly increases with applied pressure due to compressibility

$$\alpha = \alpha_0 \left(1 + \frac{\Delta P}{P_a}\right), \tag{S5}$$

where α_0 is the resistance at zero applied pressure and P_a is the pressure at which the resistance doubles. Finally, their cake build-up is described by

$$\frac{d\omega}{dt} = (J - J_{ss} h(\omega)) c_b, \tag{S6}$$

i.e. by the mass balance based on the permeate flux J, the back transport flux, and the bulk concentration c_b. The back-transport flux is the product of the steady-state flux J_{ss} and the function $h(\omega)$

$$h(\omega) = 1 - e^{-\frac{\omega}{\omega_c}}, \tag{S7}$$

with ω_c interpreted as the value of the cake mass at the steady state. This interpretation is valid, though, only for highly compressible cakes (not our case).

S3.2. Supplementary Note 7

Table S4: Symbols and units (c. - coefficient; f. factor; star * indicates SI unit). $\alpha_0^*(=\alpha_0)$ given as a range, its value determined by experiments, Table S5. 'Our' units refer to the units used in this study.

SI Units*	Symbol	Our Units	Name
[m^{-1}]	$R=\mu k_0 R^*/A^*$	[bar h L^{-1}]	resistance
[Pa]	ΔP	[bar]	TMP
[Pa]	P_a	[bar]	compress. c.
[kg m^{-2}]	$\omega=\mu k_0\alpha_0^*\omega^*/A^*$	[bar h L^{-1}]	cake thick.
[ms^{-1}]	$J=k_1 A^* J^*$	[L h^{-1}]	flux
[kg m^{-3}]	$c_b=\mu k_0^2\alpha_0^* c_b^*/A^{*2}k_2$	[bar h L^{-2}]	bulk conc.
[$kgm^{-2}s^{-1/2}$]	$\tilde{\sigma}=\mu k_0\sqrt{k_2}\alpha_0^*\tilde{\sigma}^*/A^*$	[bar $h^{1/2}L^{-1}$]	diff. c.
[$s^{-1/2}$]	$\sigma_0=\sqrt{k_2}\sigma_0^*$	[$h^{-1/2}$]	diff. c.
[Pa s]	$\mu=(1/3.6)\cdot 10^{-11}$	[bar h]	dyn. visc.
[m^2]	$A^*=5\cdot 10^{-3}$	[m^2]	memb. surf.
[m kg^{-1}]	$\alpha_0^*\sim 10^9-10^{13}$	[m kg^{-1}]	spec. resist.
	$k_0=10^{-3}$	L $\to$ m^3	conv. f.
	$k_1=3.6\cdot 10^6$	m^3h $\to$ Ls	conv. f.
	$k_2=3600$	h $\to$ s	conv. f.

Translation between our and SI units is accomplished by comparing model equations in both unit systems, or by dimensional analysis, Table S4.

Our pressures are measured in [bar] and flow rates in [L h^{-1}]. R has the unit [bar h L^{-1}], Eq. 2, as μ is incorporated in it. To transform R to SI unit, [m^{-1}], we use, say, for the native resistance R_m (Sec. S1.1)

$$R_m^*=\frac{A^*}{k_0}\left(\frac{R_m}{\mu}\right)=\frac{A^*}{\mu k_0}R_m, \tag{S8}$$

where A^* is the membrane surface and k_0 a conversion factor, Table S4. The star indicates SI unit ($A^*=A$ and $\alpha_0^*=\alpha_0$ because of the same dimensions in both systems). Note that μ is in [bar h]. The measured R_m =0.59 [bar h L^{-1}] of Sec. S1 becomes $R_m^*=9.1\cdot 10^{11}$ [m^{-1}], incidentally almost exactly the value of $R_m^*=8.5\cdot 10^{11}$ [m^{-1}] from [13].

Eq. 4 shows that ω has the same unit as R, [bar h L^{-1}]; on the other hand, the product $\alpha_0^*\omega^*$ has dimension of R_m^*, Table S4. By analogy with Eq. S8

$$\omega^*\alpha_0^*=\frac{A^*}{\mu k_0}\omega. \tag{S9}$$

Flux J(or $\mathcal{J}$, J_{ss} and Q) is transformed as

$$J^*=\frac{1}{k_1 A^*}J, \tag{S10}$$

with k_1 another conversion factor. 1 [L h^{-1}] corresponds to 65 [μm s^{-1}] for the surface of A=42.75 cm^2.

Next, from Eq. 5 (without the diffusion term) we obtain

$$c_b^*\alpha_0^*=\frac{A^{*2}k_2}{\mu k_0^2}c_b, \tag{S11}$$

from which we see that knowing independently the concentration c_b (CTSM-R parameter) and the c_b^* (from experiments), we can determine α_0^*.

The diffusive terms are somewhat more complicated. We need to remember that the differential Wiener process

$$dW_t=\sqrt{dt}N_t^{t+dt}(0,1), \tag{S12}$$

is proportional to dt (as the unit normal (of zero mean and unit variance), $N_t^{t+dt}(0,1)$, adds both positive and negative contributions: $\sqrt{dt}N_t^{t+dt}\sim dt$), and that random walk of ω gives

$$\langle\Delta\omega^2\rangle_t=N_0^t(0,\tilde{\sigma}^2 t), \tag{S13}$$

i.e. that the mean square distance is proportional to t. The units are thus $[\tilde{\sigma}]$=$[\omega/\sqrt{t}]$=[ω $t^{-1/2}$] and $[\sigma_0]$=$[1/\sqrt{t}]$=[$t^{-1/2}$]. Note the connection $\tilde{\sigma}^2=2D$, where D is the usual diffusion coefficient of the diffusion PDE (but here with ω 'distance').

Translating $\tilde{\sigma}$ and σ_0 into $\tilde{\sigma}^*$ and σ_0^* we get

$$\tilde{\sigma}^*=\frac{A^*}{\mu k_0\sqrt{k_2}\alpha_0^*}\tilde{\sigma}, \tag{S14}$$

$$\sigma_0^*=\frac{1}{\sqrt{k_2}}\sigma_0. \tag{S15}$$

Parameters appearing in the exponential terms of e.g. Eqs. 6b and 10 are of dimensions to make the terms non-dimensional: $[\sigma_P]\sim$ [bar^{-1}], $[\mu_2]\sim$ [h^2 L^{-2}] etc.

S3.3. Supplementary Note 8

By inspecting the parameter values of Table 1 of the main article, we see that the parameters do not seem to differ much from model to model in

absolute terms. Statistical analysis provides precision, however, and the confidence intervals of, say, models $M1$ and $M6$ do not overlap. Statistically, the models and their values are different. As mentioned, parameters reflect the maximum likelihood scores; relatively, $M6$ performs best with respect to AIC and RMSE.

Some of the confidence intervals are extremely large (for ω_c for $M4 - M6$). These values are artifacts of numerical approximations of likelihood and should not be trusted. It is an indication that the parameter could be removed from the models without harm. Even for the first three models the effect of ω_c is negligible: $\omega_0/\omega_c \approx 27$ and $h \simeq 1$, Eq. 8. Thus, $J_{ss}h$ from Eq. 5, corresponding to the back transport, is equal to J_{ss}.

The small value of ω_c, defined in, Eq. 8, provides a new interpretation of the parameter (compare Eq. S7): it is probably a compressibility-related relaxation parameter of the cake while the cake is reaching the steady state, i.e. it is the size of the compressible part of the cake. The negligible ω_c, in contrast to that of [13], hints that our cake may be largely incompressible.

Indeed, the compressibility parameter P_a is constant in the first four models, and equal to 11.6 bar; since our maximum pressure values are 2.5 bar, by Eq. 4 the compressibility $\Delta P_{\max}/P_a$ reaches $\sim$ 20%, in contrast to [13] where it is $\sim$ 20 times larger. This is to be expected from the mixture of solids in our lakewater, see Table S1. For the last two models, P_a is a function that increases with the total volume ($M5$) or cake thickness ($M6$) (not shown).

An additional confirmation comes from the initial value for the cake thickness ω_0 that differs from zero in all of our models. We could not get the convergence for $\omega_0 \simeq 0$. This is indicating that at least part of the cake is formed very quickly, in compliance with the multiple types of ingredients - and their different time-scales of settling - in the mixture.

In our approach we do not explicitly account for the osmotic pressure; as pointed in [3], it is implicitly taken into account via the resistance R_{ct}, Eq. 2. Our parameters $(\omega_0, \omega_c, P_a)$, however, indirectly quantify aspects of the osmotic pressure and the concentration polarization.

Table S5: Selected physical parameters obtained in this study (the mean values).

Param.	Value	Units	Note
R_m^*	$9.1 \cdot 10^{11}$	[m^{-1}]	
P_a^*	$1.2 \cdot 10^{3}$	[kPa]	
$\Delta P_{\max}/P_a^*$	$2.2 \cdot 10^{-1}$		
ω^*	$1.6 \cdot 10^{-2}$	[kg m^{-2}]	ω=1 [bar h L^{-1}]
$\omega^* A^*$	$6.9 \cdot 10^{-2}$	[g]	
$d\omega^*/dt^*$	$4.5 \cdot 10^{-6}$	[kg m^{-2} s^{-1}]	
J^*	$6.5 \cdot 10^{-5}$	[m s^{-1}]	J=1 [L h^{-1}]
c_b^*	$3.4 \cdot 10^{-1}$	[kg m^{-3}]	experiments
α_0^*	$9.5 \cdot 10^{13}$	[m kg^{-1}]	
$\tilde{\sigma}^*$	$1.9 \cdot 10^{-6}$	[kg m^{-2} s$^{-1/2}$]	
σ_0^*	$1.2 \cdot 10^{-4}$	[s$^{-1/2}$]	
D^*	$3.4 \cdot 10^{-11}$	[g^2 s^{-1}]	$(\tilde{\sigma}^* A^*)^2/2$

The parameters σ_P and σ_Q of Eq. 6b quantify dependency of diffusion on input parameters: from Table 1, $\sigma_P > 0, \sigma_Q < 0$, i.e. $\exp(\sigma_P \Delta P) > 1$, $\exp(\sigma_Q Q) < 1$, consistently across the models. This means that the actual diffusion (and/or uncertainty) is more pronounced with the pressure and less so with the cross-flow than what models suggest. Hence a systematic contribution of the cake dynamics, e.g. disregarded dependence of $J_{ss}(Q)$ on pressure, or disregarded gradients of ΔP and Q in the boundary layers, is captured in the diffusivity and not in the drift term $A(\omega)$.

Finally, we list in Table S5 the experimentally obtained physical parameters in the SI units. c_b^* is obtained from the recipe in Table S1, and α_0^* calculated from Eq. S11. We also list the diffusion coefficients which are now more informative. Incidentally, the experimental values obtained in [13] are similar to ours even though it is a different system i.e., theirs are ($0.93R_m^*$, $3.4\omega^*$ $0.1J^*$, $17.6c_b^*$, $0.16\alpha_0^*$, $9 \cdot 10^{-3}P_a^*$, $17.8\Delta P_{\max}/P_a^*$). The last two values reflect their much more compressible material in relation to ours. It is intriguing that $\Delta P_{\max}/P_a^*$ of Ref. [13] is greater than ours by almost exactly the same factor their c_b^* is greater than our c_b^*; the larger compressibility thus seems to be solely due to the larger total mass. This trend is partly captured in our most accurate model $M6$ in which the compressibility parameter is a function of the cake thickness, $P_a(\omega)$.

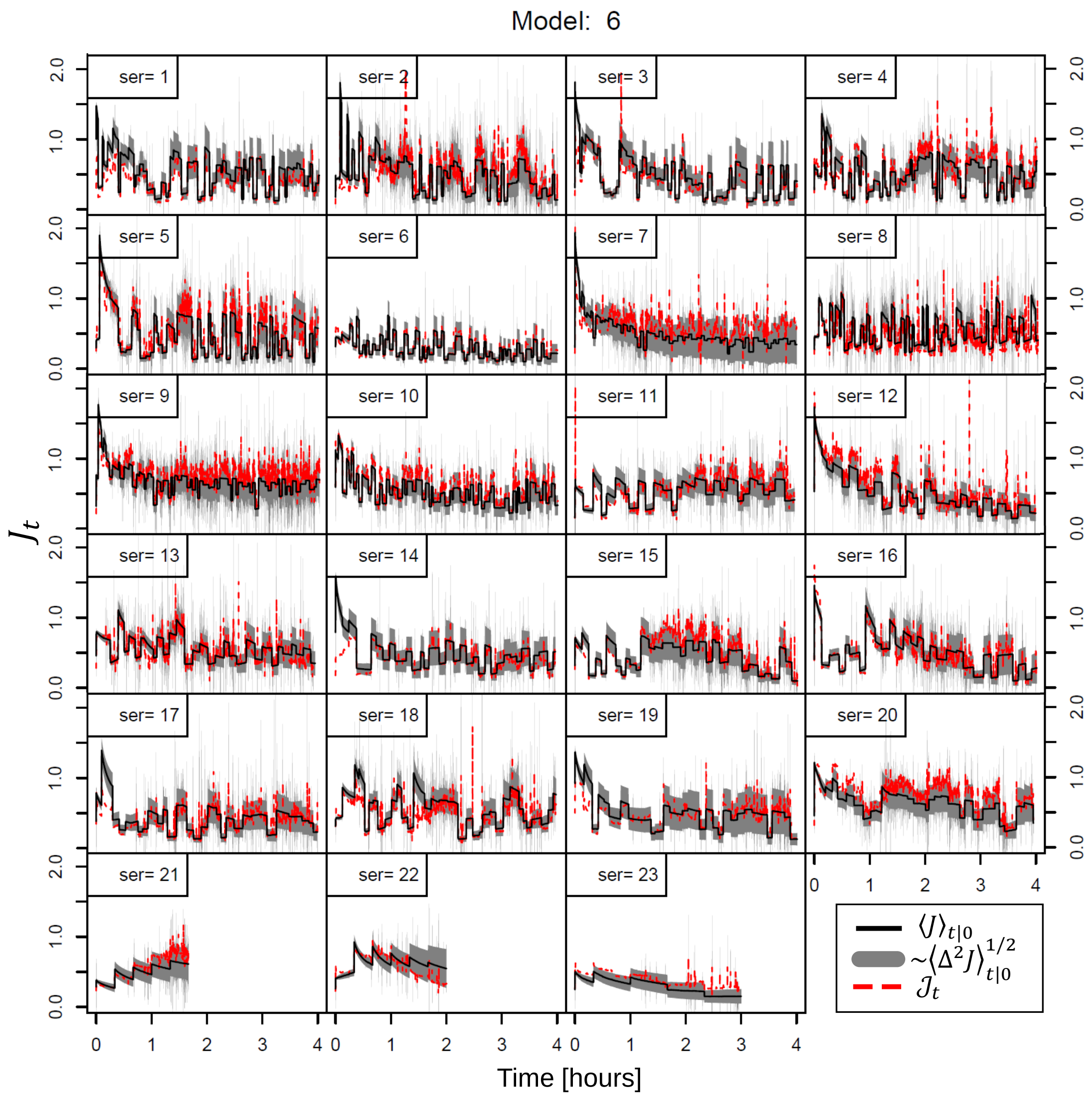

Figure S6: Flux plot, model $M6$. The legend in the bottom right corner refers to all series: $\langle J \rangle_{t|0}$ - long-term flux prediction (black), $\pm 2\sqrt{\langle \Delta^2 J \rangle_{t|0}}$ - flux prediction interval (grey), $\mathcal{J}_t$ - experimental flux (red).

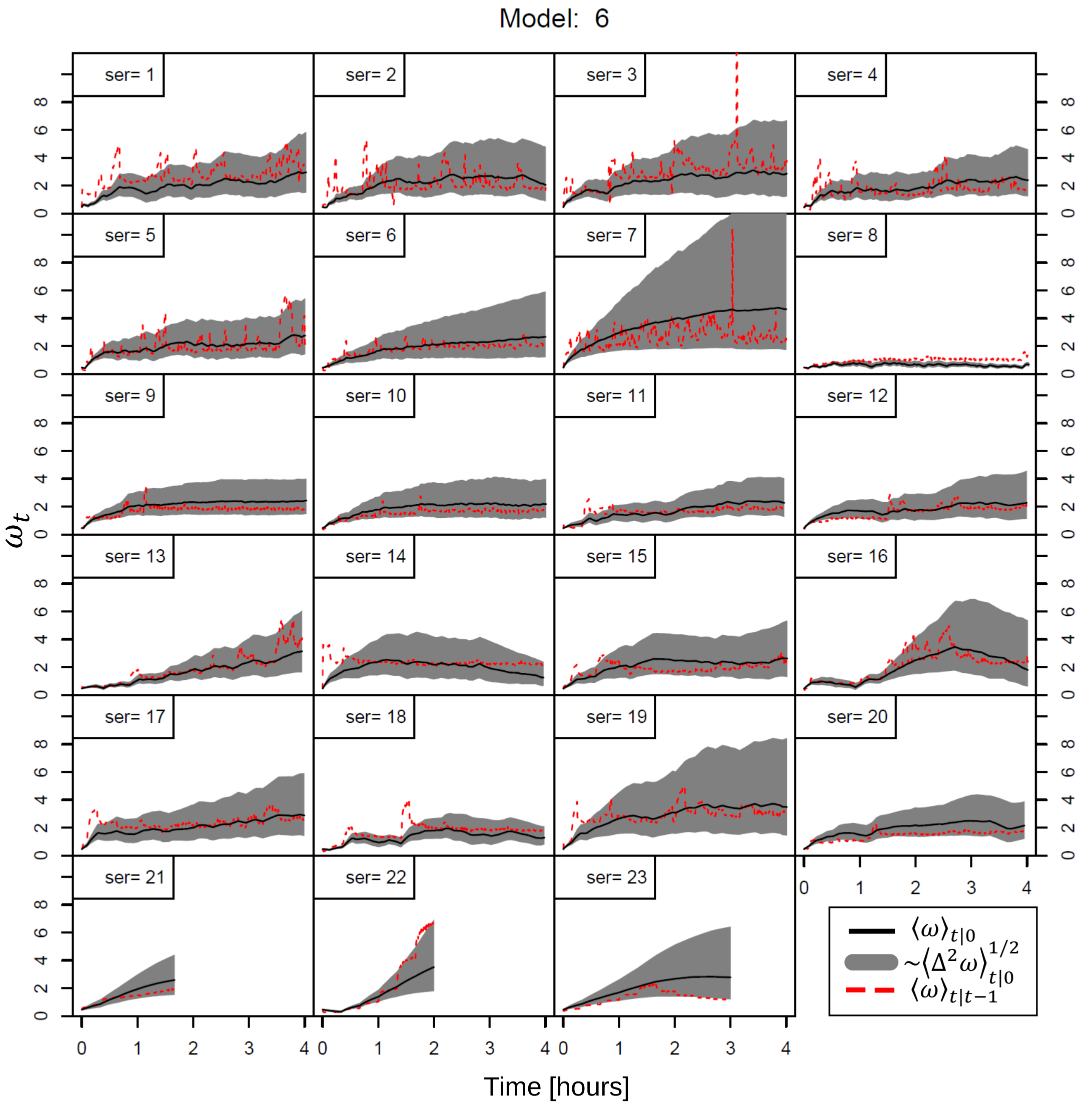

Figure S7: State plot, model $M6$. The legend in the bottom right corner refers to all series: $\langle\omega\rangle_{t|0}$ - long-term state (cake) prediction (black), $\pm 2\sqrt{\langle\Delta^2\omega\rangle_{t|0}}$ - cake prediction intervals (grey), $\langle\omega\rangle_{t|t-1}$ - reconstructed cake i.e. one-step ahead prediction (red).

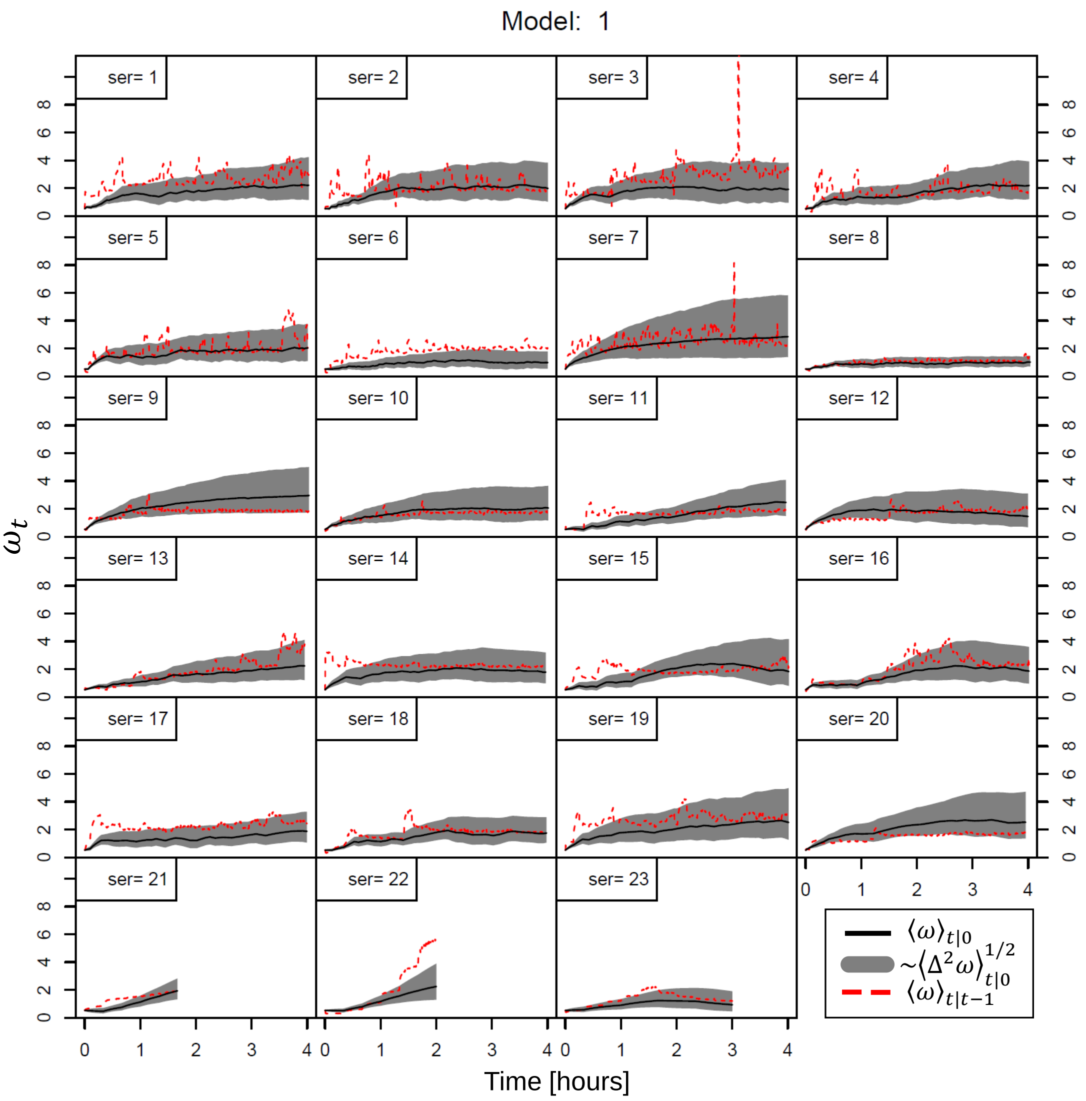

Figure S8: State plot, model $M1$. The legend in the bottom right corner refers to all series: $\langle\omega\rangle_{t|0}$ - long-term state (cake) prediction (black), $\pm 2\sqrt{\langle\Delta^2\omega\rangle_{t|0}}$ - cake prediction intervals (grey), $\langle\omega\rangle_{t|t-1}$ - reconstructed cake i.e. one-step ahead prediction (red).

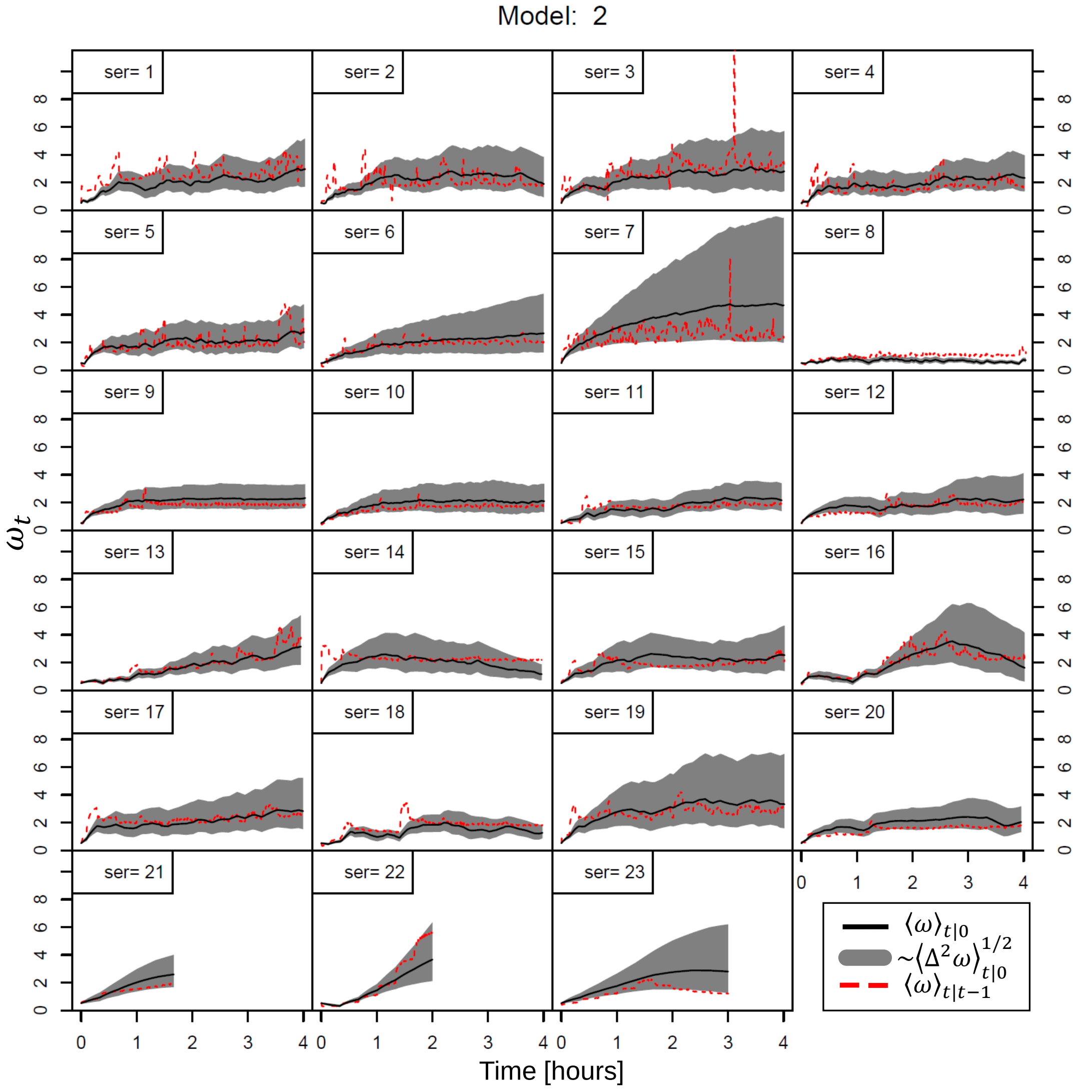

Figure S9: State plot, model $M2$. The legend in the bottom right corner refers to all series: $\langle\omega\rangle_{t|0}$ - long-term state (cake) prediction (black), $\pm 2\sqrt{\langle\Delta^2\omega\rangle_{t|0}}$ - cake prediction intervals (grey), $\langle\omega\rangle_{t|t-1}$ - reconstructed cake i.e. one-step ahead prediction (red).

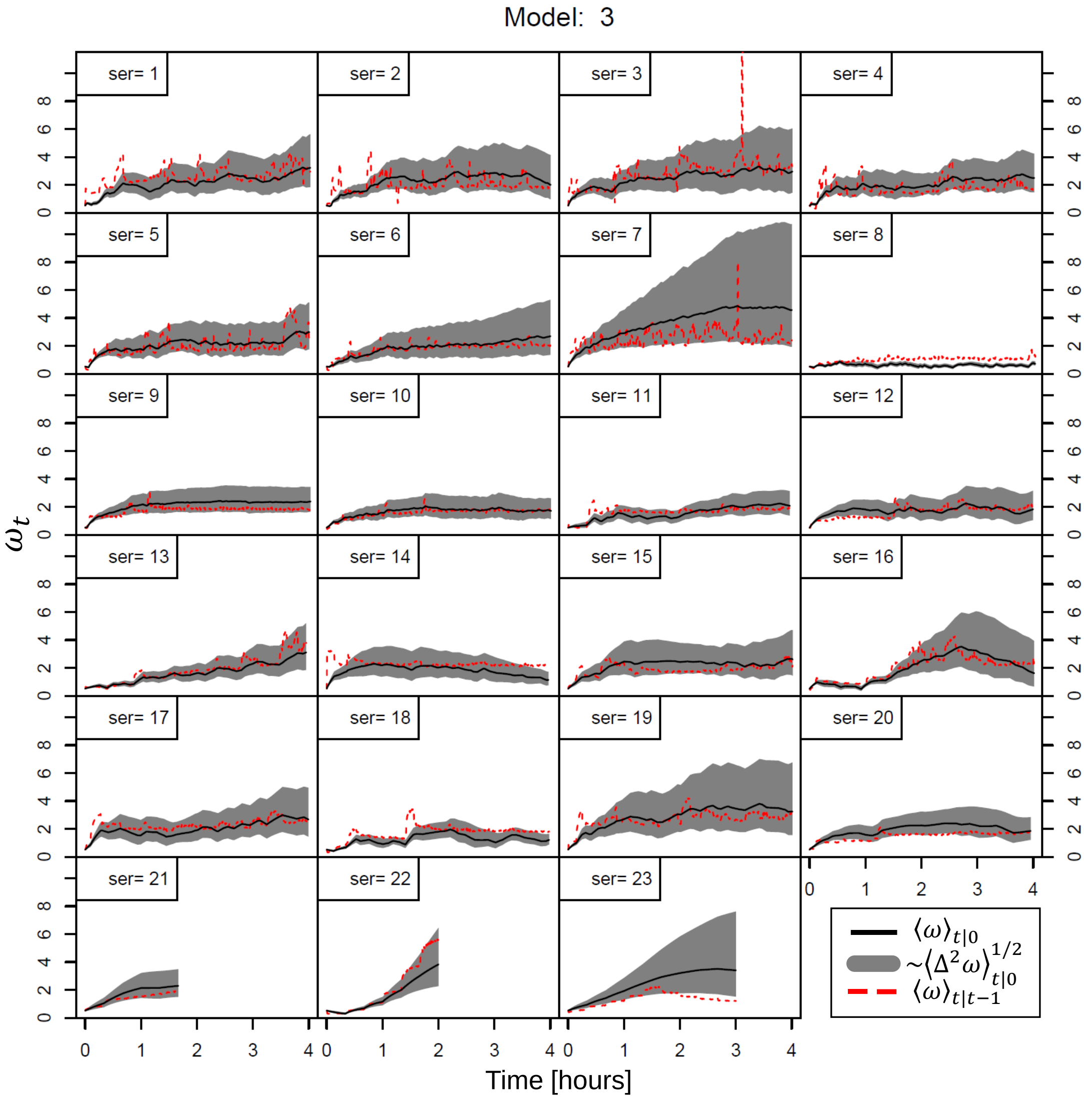

Figure S10: State plot, model $M3$. The legend in the bottom right corner refers to all series: $\langle\omega\rangle_{t|0}$ - long-term state (cake) prediction (black), $\pm 2\sqrt{\langle\Delta^2\omega\rangle_{t|0}}$ - cake prediction intervals (grey), $\langle\omega\rangle_{t|t-1}$ - reconstructed cake i.e. one-step ahead prediction (red).

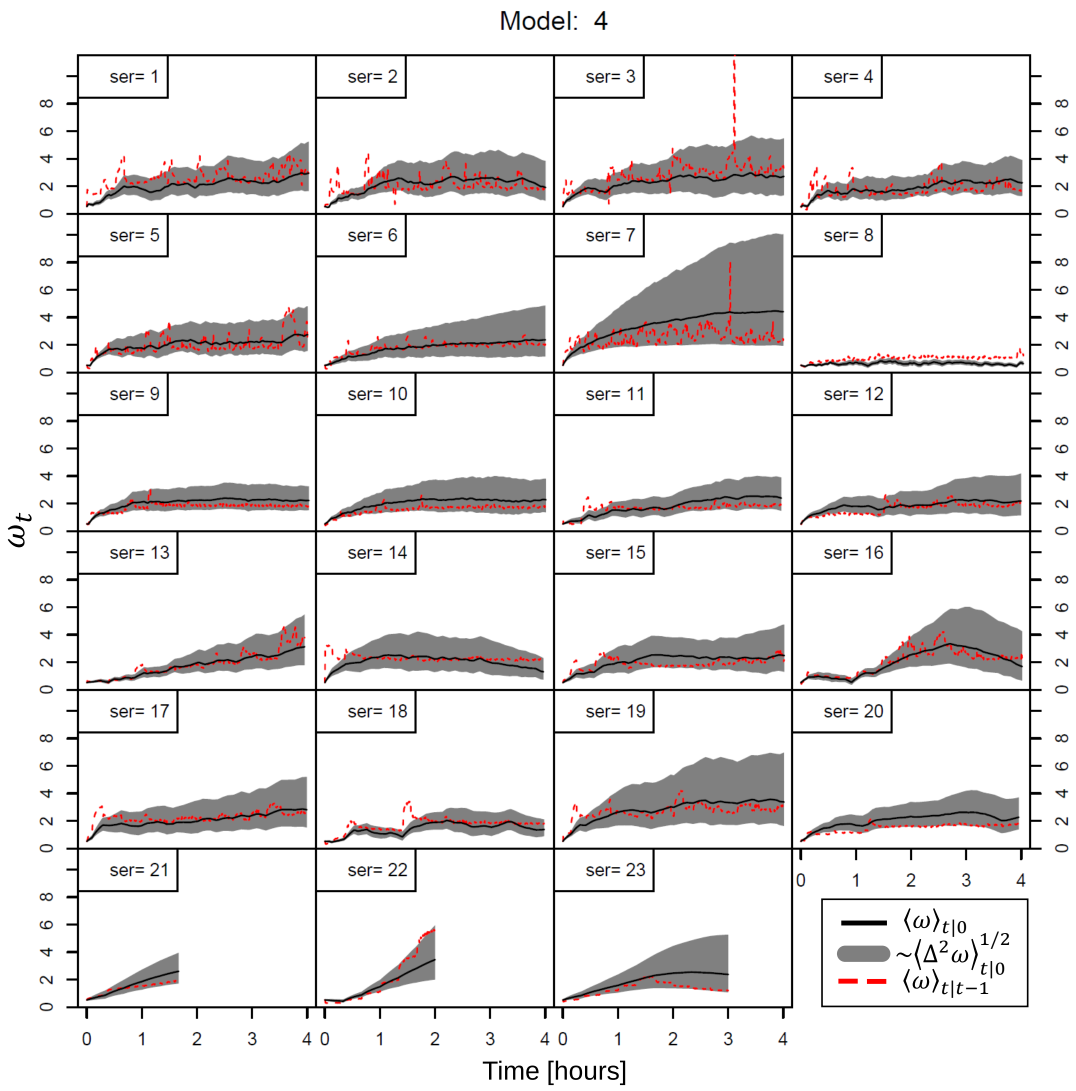

Figure S11: State plot, model $M4$. The legend in the bottom right corner refers to all series: $\langle\omega\rangle_{t|0}$ - long-term state (cake) prediction (black), $\pm 2\sqrt{\langle\Delta^2\omega\rangle_{t|0}}$ - cake prediction intervals (grey), $\langle\omega\rangle_{t|t-1}$ - reconstructed cake i.e. one-step ahead prediction (red).

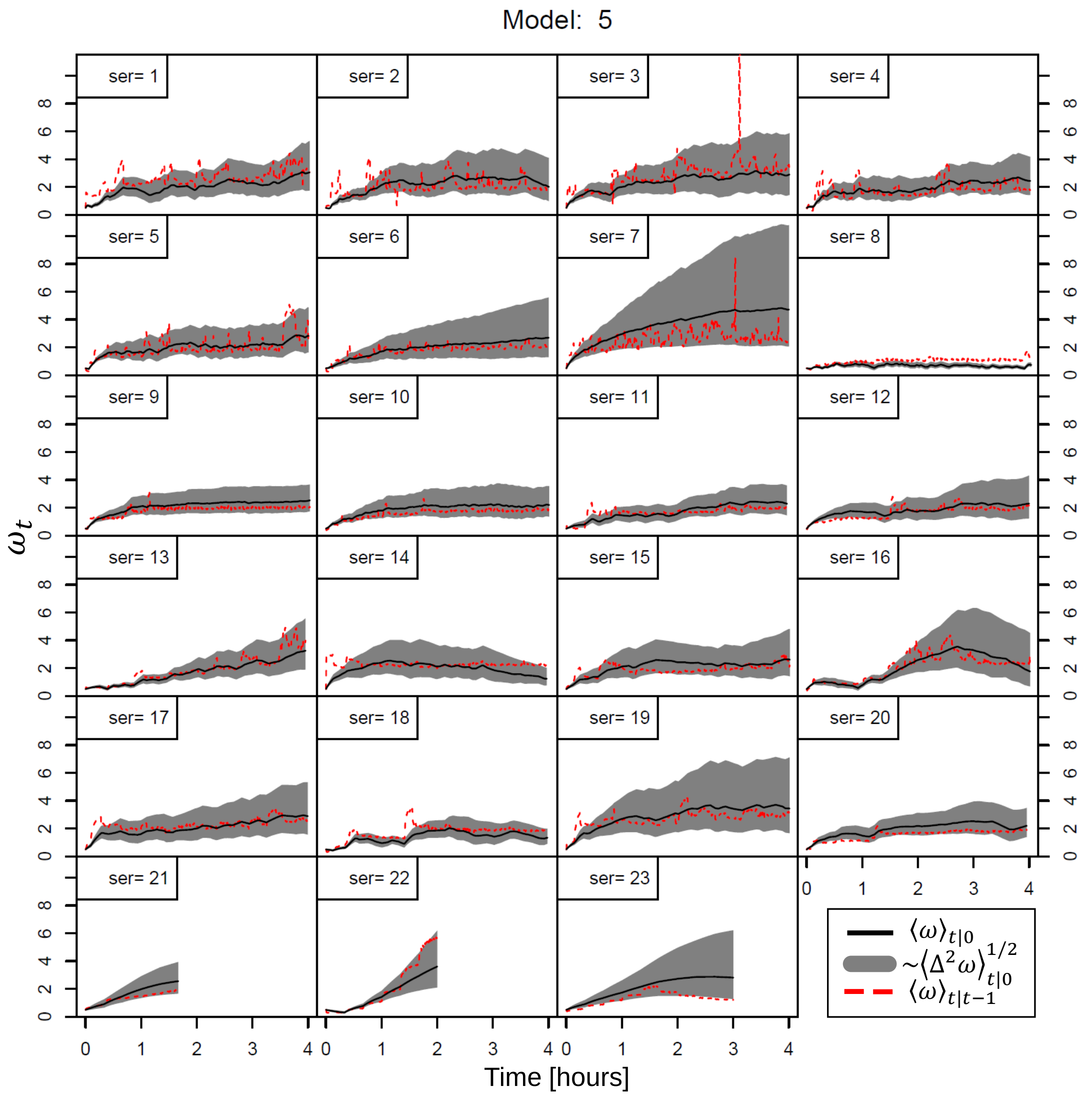

Figure S12: State plot, model $M5$. The legend in the bottom right corner refers to all series: $\langle\omega\rangle_{t|0}$ - long-term state (cake) prediction (black), $\pm 2\sqrt{\langle\Delta^2\omega\rangle_{t|0}}$ - cake prediction intervals (grey), $\langle\omega\rangle_{t|t-1}$ - reconstructed cake i.e. one-step ahead prediction (red).

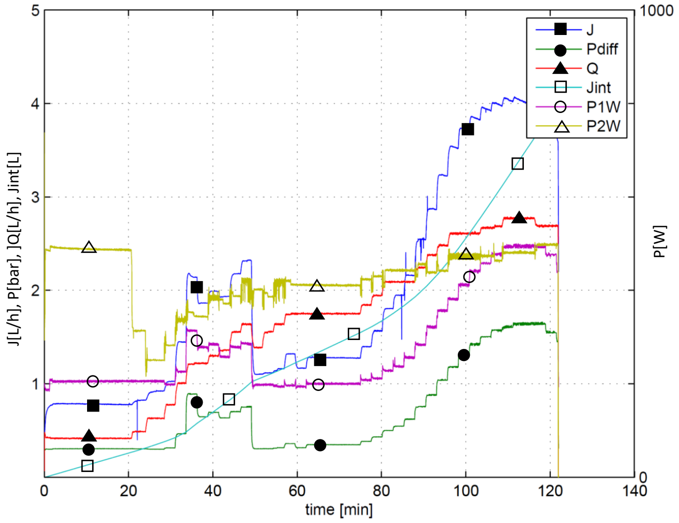

Figure S13: A test control run. 4 L of filtered water are obtained over 2 h period under time-dependent pressure and cross-flow, but without energy optimization. Note that the consumed power of the cross-flow pump (P2W, in yellow, empty triangles) is higher than the power of the pressure pump (P1W, violet, empty circles), almost throughout the entire period.

S3.4. Supplementary Note 9

Fluxes and states of the best model $M6$ vs. the experimental counterparts, for all 23 series, are shown in the Figs. S6 and S7, respectively. The states of the other five models for all series are shown in Figs. S8 - S12.

The red lines in the flux graphs are the actual experiments $\mathcal{J}_t$. The red lines in the state graphs are the one-step ahead predictions $\omega_{k|k-1}$ (actually $\omega_{t|t-1}$ as t is continuous), i.e. the cake thicknesses updated on the measurements $\mathcal{J}_k$ and closest one gets to the actual behavior of the cake in the absence of direct measurements.

Model $M1$ has the thinnest prediction intervals (in grey) and accordingly the worst fitting performance of all the models.

S3.5. Supplementary Note 10

As mentioned, we optimized for the least energy consumption associated with the cross-flow under the constraint of filtering constant volume of water. Although both pressure and cross-flow contribute to energy consumption, the cross-flow power was generally larger than that of the pressure, as seen in Fig. S13 where the control was tested without energy optimization.

S3.6. Supplementary Note 11

From Eqs. 1 and 19a (which we write again for easier following)

$$\mathcal{J}_k = J_k + e_k,$$
$$\langle J\rangle_{k|k-1} = C\langle\omega\rangle_{k|k-1},$$

and the definition of variance, we get Eq. 19b

$$\begin{aligned}\langle\Delta^2 J\rangle_{k|k-1} &\equiv \langle(\mathcal{J}_k - \langle J\rangle_{k|k-1})^2\rangle = \\ &= \langle(C\omega_k - C\langle\omega\rangle_{k|k-1} + e_k)^2\rangle \\ &= \langle(C(\omega_k - \langle\omega\rangle_{k|k-1}) + e_k)^2\rangle \\ &= C^2\langle\Delta^2\omega\rangle_{k|k-1} + S_k,\end{aligned} \tag{S16}$$

where ω_k is the true value, and we used the mutual independence of e_k and $(\omega_k - \langle\omega\rangle_{k|k-1})$, i.e. of the measurement- and the process randomness.

Similarly, from the innovation equation

$$\epsilon_k = \mathcal{J}_k - \langle J\rangle_{k|k-1},$$

and the above Eqs. 1 and 19a, Eq. 20a becomes

$$\begin{aligned}\langle\omega\rangle_{k|k} &= \langle\omega\rangle_{k|k-1} + K_k\epsilon_k, \\ &= \langle\omega\rangle_{k|k-1} - K_kC\langle\omega\rangle_{k|k-1} + K_k(C\omega_k + e_k)\end{aligned}$$

which we use to get

$$\begin{aligned}&\langle\Delta^2\omega\rangle_{k|k} \equiv \langle(\omega_k - \langle\omega\rangle_{k|k})^2\rangle = \\ &= \langle(\omega_k - K_kC\omega_k - (1-K_kC)\langle\omega_{k|k-1}\rangle - K_ke_k)^2\rangle \\ &= \langle((1-K_kC)\omega_k - (1-K_kC)\omega_{k|k-1} - K_ke_k)^2\rangle \\ &= \langle((1-K_kC)(\omega_k - \langle\omega\rangle_{k|k-1}) - K_ke_k)^2\rangle \\ &= (1-K_kC)^2\langle\Delta^2\omega\rangle_{k|k-1} + K_k^2S_k,\end{aligned} \tag{S17}$$

where again we took advantage of the mentioned mutual independence. Applying the extremum condition $\partial/\partial K = 0$ to Eq. S17, we obtain Eqs. 20b and 20c.

Note that Eq. 20b can be put in still another format

$$\frac{1}{\langle\Delta^2\omega\rangle_{k|k}} = \frac{1}{\langle\Delta^2\omega\rangle_{k|k-1}} + \frac{1}{\frac{1}{C^2}S_k}, \tag{S18}$$

which is the parallel resistance relationship, easy to memorize. In other words, $\langle\Delta^2\omega\rangle_{k|k}$ is smaller than either $\langle\Delta^2\omega\rangle_{k|k-1}$ or $\frac{1}{C^2}S_k$.

Supplementary References

[1] P. A. Stentoft, T. Munk-Nielsen, L. Vezzaro, H. Madsen, P. S. Mikkelsen, J. K. Møller, Towards model predictive control: Online predictions of ammonium and nitrate removal using stochastic asm, Water Science and Technology 79 (1) (2019) 51–62.

[2] R. F. Probstein, Physicochemical Hydrodynamics: An Introduction, Second Edition, 2nd Edition, John Wiley & Sons, Inc., 1994.

[3] R. Field, Fundamentals of fouling, in: Membranes for Water Treatment: Volume 4. Eds. Klaus-Viktor Peinemann and Suzana Pereira Nunes, WILEY-VCH Verlag GmbH & Co. KGaA, Weinheim, 2010, pp. 1–23.

[4] H. Madsen, J. Holst, P. Thyregod, A continuous time model for the variations of air temperature, in: 10th Conference on Probability and Statistics in Atmospheric Science, American Meteorological Society, Edmonton, 1987, pp. 52–58.

[5] J. L. Jacobsen, H. Madsen, Grey box modelling of oxygen levels in a small stream, Environmetrics 7 (1996) 109–121.

[6] J. K. Møller, K. R. Bergmann, L. E. Christiansen, H. Madsen, Development of a restricted state space stochastic differential equation model for bacterial growth in rich media, Journal of Theoretical Biology 305 (2012) 78–87. doi:10.1016/j.jtbi.2012.04.015.

[7] R. Kalman, A new approach to linear filtering and prediction problems, Transactions of the ASME–Journal of Basic Engineering 82, Series D (1960) 35–45.

[8] R. Kalman, R. Bucy, New results in linear filtering and prediction theory, Transactions of the ASME–Journal of Basic Engineering 83, Series D (1961) 95–108.

[9] A. H. Jazwinski, Stochastic Processes and Filtering Theory, Academic Press, New York, USA, 1970.

[10] S. Chandrasekhar, Stochastic problems in physics and astronomy, Reviews of Modern Physics.

[11] G. E. Uhlenbeck, L. S. Ornstein, On the theory of brownian motion, Physical Review 36 (1930) 823–41.

[12] CTSM-R Development Team, Continuous Time Stochastic Modelling in R, User's Guide and Reference Manual (2015).
URL `http://ctsm.info/`

[13] T. V. Bugge, M. K. Jørgensen, M. L. Christensen, K. Keiding, Modeling cake buildup under TMP-step filtration in a membrane bioreactor: Cake compressibility is significant, Water Research 46 (14) (2012) 4330–4338. doi:10.1016/j.watres.2012.06.015.
