# Supplementary material for: A data-driven digital twin for water ultrafiltration
Source: Commun Eng. 2022 Sep 30;1:23. doi: 10.1038/s44172-022-00023-6 (PMC10956058; doi:10.1038/s44172-022-00023-6)
Supplement: Supplementary file 1 — Goranovic_PR file [file 44172_2022_23_MOESM1_ESM.pdf]

nature portfolio

Peer Review File

A data-driven digital twin for water ultrafiltration

Open Access This file is licensed under a Creative Commons Attribution 4.0 International License, which permits use, sharing, adaptation, distribution and reproduction in any medium or format, as long as you give appropriate credit to the original author(s) and the source, provide a link to the Creative Commons license, and indicate if changes were made. In the cases where the authors are anonymous, such as is the case for the reports of anonymous peer reviewers, author attribution should be to 'Anonymous Referee' followed by a clear attribution to the source work. The images or other third party material in this file are included in the article's Creative Commons license, unless indicated otherwise in a credit line to the material. If material is not included in the article's Creative Commons license and your intended use is not permitted by statutory regulation or exceeds the permitted use, you will need to obtain permission directly from the copyright holder. To view a copy of this license, visit http://creativecommons.org/licenses/by/4.0/.

Reviewers' comments:

Reviewer #1 (Remarks to the Author):

In this manuscript, Stochastic Greybox Modeling and Control is a digital-twin methodology that predicts mean values and variances of (hidden) physical states, given the uncertain observations of functions of these states. Moreover, these data can reconstruct the states, providing statistical measures and proving very useful in real-world . The manuscript could be considered for publication if the following issues could be addressed

1. Can the author explain why using the fixed control instead of ratio control.

2. In sections 2.4, why you don,t use more data in statistical validation but 23 datas.

3. In the whole manuscript, it is observed that many words are italicized. However, the intention for it is not mentioned, thus confusing readers.

4. In sections 2.1 to 2.4, the author mainly discussed the experimental set-up and the equation models used in the simulation. Hence, it is inappropriate to include this in the Results section.

5. In section 4.1, it is mentioned that the sampling frequency is lowered to 0.2 Hz from the original 1 Hz. This will allow easier handling of data, however, will this causes any effect on the final results of data as only average data is considered.
6. In Figure S3, the author should state clearly which color corresponds to which series and the time interval of every plot to provide readers a quick understanding of this figure through direct observation.
7. Can the author explain why only choosing series 7 to 9 for results discussion rather than other series? Is there any specific reasons?
8. In Figure 2, the cake reconstruction measurement of series 8 is mainly outside the prediction intervals. Can the author explain why Model 6 only fails to apply to series 8 but succeed in the others?

Reviewer #2 (Remarks to the Author):

The present article entitled "A data-driven digital twin of water filtration" deals with an interesting approach to model and control cross flow ultrafiltration performances. The article aims at using a Stochastic Greybox modeling and control (SGMC) using the CTSM-R package.
Reviewer is not enough skilled in stochastic greybox modeling and continuous time stochastic modeling to honestly evaluate this work. A specialist of data driven model, time series analysis… is needed to fully evaluate this study. In addition, there is too many important information given in supplementary data which oblige the reader to continuously refer to sup data which not help the understanding.
Reviewer is more used to ultrafiltration experiment and filtration performances evaluation. Consequently, I have some few comment on the article :
Introduction : Line 3 : Please remove 'usually water' as filtration is widely used in other applications than water treatment. As example, milk, wine, blood, juice…
Line 4 : The boundary pore size need to be rewritten. RO <1 nm, NF : 1-2 nm, UF : 2-100 nm, MF : 100nm – 10µm. NF cannot be classified as ~1 µm pore size ….
Line 10 : There is another important phenomena called polarization concentration that need to be assessed. Authors only refer to cake layer formation or pore blockage.
Second column, Line 5 : I don't understand why the sentence is restricted to particles larger than 100µm. Is author refer to Ergun equation or resistance in series model? The additional resistance

(called resistance in series model) could give a linear variation of permeate flux against pressure if the fouling layer is incompressible whatever the particle size. Also, for highly permeable materials (such as granular filtration) Ergun equation might be used where the flux is not linear to the pressure gradient (for turbulent regime…
Section S1 : Set-up, experimental design.
Please given the dimension and geometry (height, length) of the polymer flow chamber or at least the hydraulic diameter of the chamber. Consequently, the Reynolds number range should be given. All test were done in laminar, turbulent cross flow regime ?
The membrane resistance (Rm is usually expressed in m-1 not in bar h L-1 in order to remove temperature effect due to water viscosity). Please indicate the temperature range of the experiment. Also, please specify if a new membrane was used at every experiment. We can see that a backwash is possible on the filtration pilot. Is it used to clean the membrane between trials ? Consequently, please add standard deviation on membrane resistance (Rm =0.25 +/- ?? bar.h.L-1). The membrane permeability is about 800 +/- ?? L/H/bar/m2 at ??°C usually commercial membranes have approximately 20-30 % deviation. Otherwise the used membrane cannot be compared.
Section 1.2 : Alginate is misspelled, is it sodium alginate (Protein is bovine serum albumin (BSA), please specify the MW.
Please specify which humic acid was used. Commercial aldrich ? what is the MW ?
Kaolinite is not a salt, it is clay particle !
What is the particle size (10 to 10 µm ?) ? Please indicate the concentration of every foulant surrogates in the feed tank (75 L).
How the bulk concentration was calculated ? It is impossible to recalculate the written value (1.45 g/L). gram of what ? there is particle (kaolinite) soluble compounds (protein, alginate, humic…)…How this was obtained?
End of S1.1 : authors specify that the particles size is ranged from 10 nm to 10 µm. according to the feed suspension recipe given in section 1.2, authors refer only to the kaolinite particle? This has to be changed. The synthetic lake water has to be characterize with proper tools (HPLC-SEC, UV, turbidty, organic carbon, fluorescence, PSD…).
Section 2 results :
Also permeate quality has to be evaluated, what is the rejection ? Is the membrane reject all species (protein, alginate, humic, kaolinite…) ? what is the impact of tMP, crossflow rate on the rejection ? There is no clear proof of the membrane rejection or fouling (cake layer formation). Everything observed might be due to concentration polarization effect. In the legend of figure S1, authors specify that the system keep the particle concentration constant ?? how ? if there is fouling (cake layer formation) the colloids, particles and soluble compounds deposit on the membrane and the remaining water only circulated through a fouled membrane… Consequently, whatever the TMP or crossflow used, the permeate flux will stabilize on the value of the fouled membrane (with a lower permeability). From reviewer mind this point is crucial in order to validate the modeling. The mass balance has to be done, a critical flux measurement has to be assessed in order to fully demonstrate that irreversible fouling might be formed… In figure S2, the data series 21-23 might be probably used to evaluate the critical flux.
In addition, the justification of the rapid variation of cross-flow rate and TMP has to be given by authors. It is quite awkward to constantly vary the cross flow rate, the TMP… Usually one pressure is used for different flow rate. I don't understand why authors decided to used such noised on TMP and cross flow rate…

Finally, I cannot evaluate this article as the main originality relies on the modeling using stochastic greybox which I am not enough skilled.

Reviewer #3 (Remarks to the Author):

In this manuscript, the authors creatively proposed a digital twin methodology named Stochastic Graybox Modelling and Control (SGMC) to approximate the water filtration process. Specifically,

inheriting the core idea behind the digital twin, the physical laws and theories that represent physical system dynamics are used as the skeleton of the digital twin model, while the stochastic modelling methods are used to quantify the associated uncertainties, which together form the so-called SGMC. Regarding its application in modelling membrane filtration, the parameterisation for six typical models is given, and the (Extended) Kalman filtering is used to reconstruct the physical states of the system. The learned digital twin model then serves as the basis for better control of the filtration process. This is an interesting case of using digital twin in engineering practice, balancing data and physical knowledge and accomodating uncertainties that exist in practice. A few revisions are suggested:

1. In the concluding discussion (Sec. 3), the authors mentioned that SGMC applies to some real-world settings, such as wastewater treatment plants or wind-energy production. It would be beneficial if the authors can specify the potential application conditions. For example, is it applicable to all processes that can be modelled using deterministic ODE?
2. Figure S3 seems a little bit confusing. The authors are suggested to find a better diagram form and give more details about the figure.

Response to Reviewers

June 1, 2022

Below are our detailed responses to the three reviewers. We thank them for the detailed feedback that we believe have led to significant improvements of our article. To facilitate reading this document we have highlighted our own responses in blue. Similarly, all updates to our article are highlighted in blue.

1 Reviewer 1

In this manuscript, Stochastic Greybox Modeling and Control is a digital-twin methodology that predicts mean values and variances of (hidden) physical states, given the uncertain observations of functions of these states. Moreover, these data can reconstruct the states, providing statistical measures and proving very useful in real-world . The manuscript could be considered for publication if the following issues could be addressed

1. Can the author explain why using the fixed control instead of ratio control.

This was just our choice. We optimize for the least energy consumption associated with the cross-flow that controls the cake, under the constraint of obtaining a fixed volume of water. Such a scenario could be relevant in preexisting industrial operations where delivery of fixed amount of filtered solvent needs to be automated under minimal cost. The last sentence was now added in the main text in Sec. 2.7. The ratio control, in which input-to-output ratio is kept constant was not a problem to implement.

2. In sections 2.4, why you don,t use more data in statistical validation but 23 datas.

We are not sure what is meant by '23 datas'. We use 23 data sets: there were in total about 89 hours of measurement and hence roughly 89*3600/5=64000 data-points for flux, pressure and cross-flow distributed over the 23 data-series. All these points are used for statistical analysis. This exceeds substantially the ordinary measurements under constant pressure/cross-flow, which are on the order of 10 data points for the input data (e.g. a fixed pressure and a few variable cross-flows). The particular choice of the 23 input sets was governed by the random experimental design as explained in Methods, Sec. 4.1, and available lab-time. We have now better emphasized the number of data points in Sec. 2.4.

3. In the whole manuscript, it is observed that many words are italicized. However, the intention for it is not mentioned, thus confusing readers.

We have removed most of the italicized words (there were 16 in total), retaining only five: for the emphasis of the three set of equations in Kalman filtering method (Sec. 4.2), and for the self-explanatory words '*exactly*' (in relation to the exactness of the Kalman filtering), and '*hidden*' (in relation to the non-observed states). In addition, we offered brief contextual explanations

in two cases. For example, the phrase 'our methodology features *modeling* of uncertainties' is changed to 'our methodology features actual modeling of uncertainties.'

4. In sections 2.1 to 2.4, the author mainly discussed the experimental set-up and the equation models used in the simulation. Hence, it is inappropriate to include this in the Results section.

Sec. 2.1-2.4 are all building-up the model identification which is one of the main results of our article. In Sec 2.1, the set-up is just briefly mentioned, the emphasis being on our novel experimental design and randomized data. We shortened the name of the Sec. 2.1 to 'Experimental design and data' to reflect that fact better. In Sec. 2.2., stochastic greybox modeling framework - not a common knowledge - is summarized via equations that we later use for modeling. In Sec 2.3., our actual models are systematically presented. They differ from a set of literature models that are conveniently shown in Sec. 3.3.1. Finally, Sec. 2.4. is featuring model parameters and statistical validations of the models. All these are results. We changed the name of the section to "Results and Discussion".

5. In section 4.1, it is mentioned that the sampling frequency is lowered to 0.2 Hz from the original 1 Hz. This will allow easier handling of data, however, will this causes any effect on the final results of data as only average data is considered.

We used the averaging mainly to prevent the instances of zero permeate flux as there were hardly any drops of water passing through membrane over very short times. This would require a more complicated statistical analysis. Our averaging still enables us to discern the two time scales in flux data, instantaneous vs. diffusing one, as well as the temporal changes of the input variables, and so does not affect our conclusions. Note that if the averaging was over a much larger period of time, in which the temporal changes of pressure and cross-flow were averaged out, we would get into the constant-input regime, typical for traditional experiments. We added a note in the Sec 4.1 about the averaging.

6. In Figure S3, the author should state clearly which color corresponds to which series and the time interval of every plot to provide readers a quick understanding of this figure through direct observation.

We have removed the old Figure S3 showing the visited parts of $\Delta P - Q$ space in the randomization, because its size was not justified by the content it provided. Besides, the randomized values of the pressure and cross-flow can be seen from Figure S4a. Our new Figure S3 gives model predictions for constant pressure and constant cross-flow input.

7. Can the author explain why only choosing series 7 to 9 for results discussion rather than other series? Is there any specific reasons?

The three series allow us to focus on some important aspects of the modeling results and contain almost all general aspects seen in other series. Our graphs are not typically encountered in traditional experimental (physical) sciences. Their features, nicely seen in the three series, are different-size prediction intervals i.e. standard deviations (grey areas) that replace the Monte Carlo simulations, dynamics of reconstructed cake, visible in ser. 7, and the mean values of all three series which are close to a constant, essentially attaining the steady states known from traditional types of measurements to which our results are then easier to relate. Also, comparison of the flux and hidden cake can be conveniently juxtaposed against each other for a triplet. Note that the ΔP_t and Q_t inputs of the three series are also easy to interpret, Fig.1a. Having said all this, we added in Sec. 2.5 specific comments on trends seen in other series, Fig. S7-S12.

8. In Figure 2, the cake reconstruction measurement of series 8 is mainly outside the prediction intervals. Can the author explain why Model 6 only fails to apply to series 8 but succeed in the others?

Series 8 corresponds to high cross-flow and low pressure input (on average), Fig. 1a, top middle panel. In such situation, where cake is almost removed and little additional mass is coming to membrane, the model $M6$, and in fact all other models except $M1$, overestimate somewhat the flux and underestimate the cake, Figs. S7-S12. Part of the reason is the nature of models seen in Fig. 3, which we now updated to include also model $M1$; namely, all models predict a higher (steady state) flux at high cross-flow in relation to $M1$. Situation is opposite for ser. 6 which features low pressure and low cross-flow inputs, Fig. S4a; here, $M1$ somewhat overestimates the flux and underestimates the cake and all other models get it right, see ser. 6 in Figs. S7-S12; from Fig. 3, we see that $M1$ predicts higher flux at low cross-flow in relation to other models. None of the models is perfect - hence our statistical ranking of the proposed models. The fact that a single series is not predicted correctly (within confidence intervals) by a model, corresponds to a single point outlier, say from a linear law/graph, in traditional single constant-input measurements. When there are such complex interactions as in ultrafiltration of many different molecular species, we dare to say that a mismatch is inevitable. We added a shortened version of the discussion in Sec. 2.5.

2 Reviewer 2

The present article entitled "A data-driven digital twin of water filtration" deals with an interesting approach to model and control cross flow ultrafiltration performances. The article aims at using a Stochastic Greybox modeling and control (SGMC) using the CTSM-R package. Reviewer is not enough skilled in stochastic greybox modeling and continuous time stochastic modeling to honestly evaluate this work. A specialist of data driven model, time series analysis... is needed to fully evaluate this study. In addition, there is too many important information given in supplementary data which oblige the reader to continuously refer to sup data which not help the understanding.

Reviewer is more used to ultrafiltration experiment and filtration performances evaluation. Consequently, I have some few comment on the article :

NB. All of the questions are addressed and answers incorporated into the document: We have rewritten and corrected the whole supplementary section S1 that describes the experimental system, and supplemented it with two new figures. We removed a figure from Sec. S2 and added one in Sec. S4. We moved table S3 (validation of models) into Table 1 of the main file where we also appended the sections 2.4-2.6 with relevant discussions on parameters, critical flux and osmotic pressure.

Introduction :

1. Line 3 : Please remove "usually water" as filtration is widely used in other applications than water treatment. As example, milk, wine, blood, juice...

Done. Thank you for the examples.

2. Line 4 : The boundary pore size need to be rewritten. RO <1 nm, NF : 1-2 nm, UF : 2-100 nm, MF : 100nm - 10μm. NF cannot be classified as $\approx$1 μm pore size....

Updated. Thank you.

3. Line 10 : There is another important phenomena called polarization concentration that need to be assessed. Authors only refer to cake layer formation or pore blockage.

Updated. Thank you.

4. Second column, Line 5 : I don't understand why the sentence is restricted to particles larger than 100μm. Is author refer to Ergun equation or resistance in series model? The additional resistance (called resistance in series model) could give a linear variation of permeate flux against pressure if the fouling layer is incompressible whatever the particle size. Also, for highly permeable materials (such as granular filtration) Ergun equation might be used where the flux is not linear to the pressure gradient (for turbulent regime...

We have removed the constraint and added a note for turbulent regime.

5. Section S1 : Set-up, experimental design. Please given the dimension and geometry (height, length) of the polymer flow chamber or at least the hydraulic diameter of the chamber. Consequently, the Reynolds number range should be given. All test were done in laminar, turbulent cross flow regime?

The membrane had dimensions (L, W, H)= (10 cm x 5 cm x 2 mm), and was mounted inside the chamber of inner dimensions (L, W, H)=(9.5 cm x 4.5 cm x 4 cm); the membrane's active area was thus $A \simeq 43$ cm^2. Maximum Reynolds number (velocity corresponding to $Q = 3.5$ L/h going through inlet pipes of diameter $d = 3.8$ cm) based on chamber's hydraulic diameter $D_h = 2LW/(L + W) = 6.1$ cm was $Re_{\max} = 52$, hence laminar flow.

6. The membrane resistance (Rm is usually expressed in m-1 not in bar h L-1 in order to remove temperature effect due to water viscosity). Please indicate the temperature range of the experiment. Also, please specify if a new membrane was used at every experiment. We can see that a backwash is possible on the filtration pilot. Is it used to clean the membrane between trials? Consequently, please add standard deviation on membrane resistance (Rm =0.25 +/- ?? bar.h.L-1). The membrane permeability is about 800 +/- ?? L/H/bar/m2 at ??°C usually commercial membranes have approximately 20-30 % deviation. Otherwise the used membrane cannot be compared.

Units (our vs. SI) are featured in Table (former Table S4), and converted in Sec. S3.4.2. The set-up had installed a temperature control, providing essentially the constant temperature range of $T = (22,4 \pm 0,5)$ °C; schematic of Fig. S1b was updated to include it. Backwash was not used to clean the membranes, hence we removed it from the schematic. After each experiment the membrane was chemically cleaned to remove the accumulated hardened polymer cake/gel of brownish color, and then kept submerged under water for later reuse. Altogether 14 new membranes were used.

The value $R_m = 0.25$ is erroneous (we conducted two batches of experiments and wrote the wrong value). The correct native resistance based on 14 new membranes is $R_m = (0.59 \pm 0.04)$ [bar h L^{-1}]*, and thus the permeability per unit area is $A = (40 \pm 3) \cdot 10$ [L/(h bar m^2)] at $T = (22,4 \pm 0,5)$ °C. The deviation is $\sim 7\%$ reflecting the uniformity of the German-made Inopor membranes. (*in SI unis $R_m = (9.1 \pm 0.6) \cdot 10^{11}$ [1/m].)

7. Section 1.2 : Alginate is misspelled, is it sodium alginate (Protein is bovine serum albumin (BSA), please specify the MW.

Corrected, thank you (in Danish it is spelled 'alginat'). Yes, sodium alginate with MW ranging from 12kDa to 40kDa (based on the viscosity).

8. Please specify which humic acid was used. Commercial aldrich ? what is the MW ?

Yes, humic acid was commercial Sigma-Aldrich. Being of natural origin, its composition and MW are varying from lot to lot. A true structure cannot be given since humic acid is not of uniform composition. It consists of heteropolycondensates of MWs ranging from 2,000-500,000 (but mainly 20,000-50,000).

9. Kaolinite is not a salt, it is clay particle !

Corrected, thank you.

10. What is the particle size (10 to 10 μm ?) ? Please indicate the concentration of every foulant surrogates in the feed tank (75 L). How the bulk concentration was calculated ? It is impossible to recalculate the written value (1.45 g/L). gram of what ? there is particle (kaolinite) soluble compounds (protein, alginate, humic...)...How this was obtained?

We have not measured the particle size (see Q11 below), and so we removed the numbers (they were orders of magnitude estimates based on literature values of dispersed kaolinite particles). The lake-water recipe has been updated in discussion with the responsible lab technicians. The bulk concentration was calculated as the total solid mass over the total volume of water, now being corrected to 0.34 g/L, see the updated recipe (in Q13 below we discuss more on the concentration). We emphasize that the numerical value of the bulk concentration (in SI units) serves only to get a rough estimate of the SI parameters in Table S4, namely α_0 which is unknown for our system, and few other that depend on it. We believe that there is a merit to display the (the estimates of) values of SI parameters, e.g. for orientative comparison with literature values.

11. End of S1.1 : authors specify that the particles size is ranged from 10 nm to 10 μm. according to the feed suspension recipe given in section 1.2, authors refer only to the kaolinite particle? This has to be changed. The synthetic lake water has to be characterize with proper tools (HPLC-SEC, UV, turbidty, organic carbon, fluorescence, PSD...).

Although the detailed characterization of the lake-water would have been beneficial, it was not done for two reasons: first, our focus was exclusively on models tying input sequences to flux output measurements (the only type of measurements considered), and then on predictions of these models in control algorithms for minimal energy use during the filtration process. In other words the emphasis on data-driven future forecast and not on detailed experimental analysis and related interpretation (if interpretation is possible, that is of course useful, but not necessary for real-world operations in which detailed analyses are rarely available). This precluded measurements deemed not critical for the study, as decided by the responsible project managers of the company. Their decision was governed by the second reason, namely that in all projects particularly those linked to industry, there are time and budgetary constraints; the lab work amounted to 11 full working days (normal hours) of several people, protracted over the period of four months.

12. Section 2 results : Also permeate quality has to be evaluated, what is the rejection ? Is the membrane reject all species (protein, alginate, humic, kaolinite...) ? what is the impact of tMP, crossflow rate on the rejection ?

Permeate was not measured/analyzed except for its weight, for the same reasons as mentioned in Q11 above. However, based on the inopor® product data-sheet, the cut-off for the ultrafiltration membrane with 10 nm pores is 20 kDa (under 3-8 bar). A very high percentage of the material was thus retained, especially if one considers the predominant polymer formation mediated by $CaCl_2$, visible as the brown gel on the membrane surface (see Q13).

13. There is no clear proof of the membrane rejection or fouling (cake layer formation). Everything observed might be due to concentration polarization effect. In the legend of figure S1, authors specify that the system keep the particle concentration constant ?? how ? if there is fouling (cake layer formation) the colloids, particles and soluble compounds deposit on the membrane and the remaining water only circulated through a fouled membrane... Consequently, whatever the TMP or crossflow used, the permeate flux will stabilize on the value of the fouled membrane (with a lower permeability).

As mentioned twice already, we observed at the end of each measurement that a (thin) brownish tar-like gel formed at the membrane's surface, forcing us to chemically clean the membranes after each run. The consistency of the gel was similar to the stain of evaporated coffee. The color stems likely from all three ingredients - the kaolinite, the alginate and the humic acid - with last one probably contributing the darkest hue.

We experienced that the decisive ingredient for the control of gelation was $CaCl_2$, on two accounts: 1) as a known desiccant it bounds water in the form of various hydrates e.g. $CaCl_2{\cdot}6H_2O$, and 2) Ca^{2+} ions replace sodium to cause cross-linking of different alginate chains. Type of watery gel formed is shown in Fig.1a below (in the article it is the new Fig. S2a). We had to do preliminary experiments to adjust (minimize) the amount of $CaCl_2$, since too much of it caused gelation throughout the entire piping system.

From the above discussion we can argue that our cake consisted of irreversible hard polymer mesh imbued with $CaCl_2$ hydrates and other salts which caused osmotic pressure and reversible swelling/compression of the mesh. In other words, the last two ingredients likely responded instantaneously to abrupt changes in pressure and cross-flow, making the oscillations in the flux and the cake's thickness visible from data and model predictions. However, the irreversible component was also there. Thus, both the cake and the concentration polarization contributed to lowering of the permeate flux. Our modeling results remain unaffected since the modified Darcy's resistance approach that we use in our models implicitly accounts for both (not distinguishing between the two), as shown in [R. Field, Fundamentals of fouling, in: Membranes for Water Treatment: Volume 4. Eds. Klaus-Viktor Peinemann and Suzana Pereira Nunes, WILEY-VCH Verlag GmbH & Co. KGaA, Weinheim, 2010, pp. 1-23].

Concentration was not controllably kept constant in the system (we softened the statement). As correctly shown in Fig. S1b, there is a circulation of the retentate back to the reservoir. However, only one (or at most two in a row) measurements per single filled reservoir (77 liters) were done before the rest of the water was dumped and the reservoir fully refilled with a freshly made lake-water. Because three liters were typically filtered in a measurement, the change in volume was 4-7%, but the mass of the lake-water dirt was also lowered by the amount collected in the cake. The concentration thus changed a little over the course of single measurement. As pointed by the reviewer, the composition is very complicated (inhomogeneous mixture of particles, salts and complex macromolecular species), so the number given above, 0.34 g/L, is for orientation only, to give us the feel for the magnitude of SI parameters. In real-world control scenarios, where conditions are much less controlled than in our experiments here, changes in parameters are accommodated by new parameter estimations which are re-done regularly, say, every other day, from which a new concentration can be determined. This is how a waste-water control has been effected in a plant in Denmark.

A compressed version of the answer is now included in the article.

14. From reviewer mind this point is crucial in order to validate the modeling. The mass balance has to be done, a critical flux measurement has to be assessed in order to fully demonstrate that irreversible fouling might be formed... In figure S2, the data series 21-23 might be probably used to evaluate the critical flux.

As mentioned, our models based on time-dependent Darcy's resistance are valid regardless of the mechanism of the resistance, and in fact combine both irreversible and reversible contributions. However, the critical flux is a relevant topic, and we have investigated it. The critical flux in ultrafiltration is the limiting flux reached when pressure becomes sufficiently high. Both, gel formation and concentration polarization are proposed as viable mechanisms for the phenomenon, [R. F. Probstein, Physicochemical hydrodynamics, 2003]. When gel is formed, the concentration

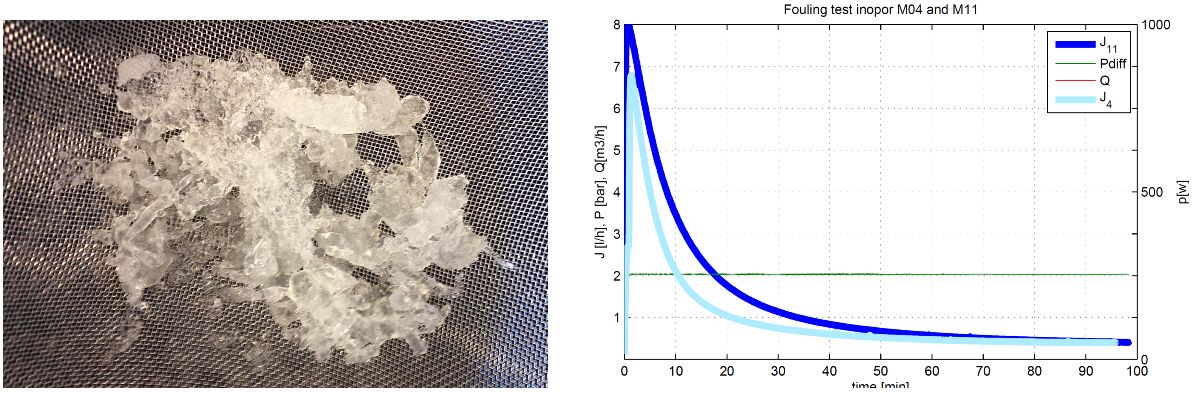

Figure 1: a) Gel formation; b) A test of fouling at constant values of pressure and cross-flow.

of accumulated filtrate reaches maximum, so upon any further increase in pressure the gel thickens rather than upconcentrates, increasing the resistance, thus lowering the flux to the previous value (the system becomes mass transfer rather than pressure dependent).

While adjusting $CaCl_2$ concentration, we performed a set of traditional constant-pressure measurements, one shown above in Fig.1b (in the article the new Fig. S2b.). Due to the cake/CP build-up, the flux stabilizes to a steady-state value, indicating the mass balance of convective and diffusive fluxes. The critical flux is the maximum steady-state flux obtainable in the system.

The steady-state flux J_{ss} explicitly enters our models, and even more, we investigated its dependence on the cross-flow, $J_{ss}(Q)$, for control purposes later on (it depends on pressure as well, but we constrained our investigations). Our models differ essentially in different functions of $J_{ss}(Q)$, see the updated Fig. 3. We see that the models predict different maximum values of J_{ss}, i.e. different Q-dependent critical fluxes, ranging from 0.41 to 0.7 L/h. Models $M1$ and $M3$ in addition offer a range of constant plateau values where J_{ss} does not change. The models of Fig. 3 represent statistically increasingly more accurate approximations of J_{ss}, see Table 1. The most accurate model $M6$ gives $J_{\text{crit}}(Q)$=0.65 L/h.

The limitation of flux (on pressure) can be inferred from e.g. ser. 7, Fig. 2. We notice that cake's prediction interval (grey area) is much larger than the corresponding one of the flux. As the system becomes mass-transfer dependent at large pressures, compatible with gel formation, the fluxes yield a much narrower range of the values, up to $\sim$ 0.7 L/h. Note that part of the uncertainty is due to σ_P that takes into account unknown aspects/dependence on pressure.

The essence of the answer is now included in the article, particularly in Sec. 2.5 and 2.6. It is important to stress that our aim was optimal control, and not the investigation of critical flux per se which might not have been even reached as our pressure did not exceed 2.5 bar. The fact that we observed a layer of cake at the end of each measurement gives us the confidence that the estimates given here are probable.

15. In addition, the justification of the rapid variation of cross-flow rate and TMP has to be given by authors. It is quite awkward to constantly vary the cross flow rate, the TMP... Usually one pressure is used for different flow rate. I don't understand why authors decided to used such noised on TMP and cross flow rate...

There are several reasons why we use time-dependent randomized input sequences of ΔP_t and Q_t.

a)The first is the model identification: we statistically probe our system in a wide range of input-output scenarios to identify model parameters to be valid across the entire range. Compared to traditional constant inputs the randomized approach is statistically more reliable - the model parameters are robust as both the choice and the number of data points is significantly larger in our case as compared to a traditional one. Strictly speaking, output data pertains only to its corresponding range of inputs, so when a model is extrapolated outside its input range, its validity there is just an assumption. Indeed, how sure can one be that models based on constant input would work for a randomized input? Our models however easily accommodate the constant input (see new Fig. S3), being thus more general.

b)The second reason is that our final goal is the control of the process, subject to predefined constraints. That requires programmability of the input sequences, akin to the randomized variations. We see in Fig. 4 that cross-flow is indeed changing rapidly to achieve the minimal energy consumption. Hence, programming the sequences for rapid random variations paves the way for programming them for any desired sequence.

c)Finally, our models are hybrids between data-driven and physical models. Our primary aim is the prediction as opposed to interpretation, since industrial operations for which the models are intended rely on limited processing time of data (interpretation is of course welcome, but not necessary). Our models thus need to rely on as wide data-driven input as possible. Randomization does that.

d)These types of inputs are our statistical novelty into the traditional field of membrane separations. Note that our variable inputs resolve the dynamics on shorter time-scales, whereas the reconstructed cakes gives us the insight into the evolution of cakes across a variety of situations (Fig. S6-S11). Both the short-time dynamics and the cakes would otherwise be completely unknown.

Essential parts of this answer append already existing points in the main text: a), b) and d) in Sec. 2.1, and c) in Introduction.

16. Finally, I cannot evaluate this article as the main originality relies on the modeling using stochastic greybox which I am not enough skilled.

We commend the referee for her/his honest self-appraisal of evaluating our primarily theoretical work that uses a novel approach. We made a conscious decision to focus on the theoretical modeling, putting thus all the experimental info into the supplementary file as it was less central to our work. Our emphasis has been on model predictions that influence the control rather than on interpretation and detailed analysis of experiments. The decision helped to shorten the article in half and bring forth our main theme - the models and the control based on them - in focus.

Sec. 2.2 (especially the newly added note), the illustrative example in Sec. S2.3, and Sec 4.2 & 4.3 of Methods are intended to give a basic primer of stochastic modeling for researchers who are not familiar with it.

3 Reviewer 3

In this manuscript, the authors creatively proposed a digital twin methodology named Stochastic Graybox Modelling and Control (SGMC) to approximate the water filtration process. Specifically, inheriting the core idea behind the digital twin, the physical laws and theories that represent physical system dynamics are used as the skeleton of the digital twin model, while the stochastic modelling methods are used to quantify the associated uncertainties, which together form the so-called SGMC. Regarding its application in modelling membrane filtration, the parameterisation

for six typical models is given, and the (Extended) Kalman filtering is used to reconstruct the physical states of the system. The learned digital twin model then serves as the basis for better control of the filtration process. This is an interesting case of using digital twin in engineering practice, balancing data and physical knowledge and accomodating uncertainties that exist in practice. A few revisions are suggested:

1. In the concluding discussion (Sec. 3), the authors mentioned that SGMC applies to some real-world settings, such as wastewater treatment plants or wind-energy production. It would be beneficial if the authors can specify the potential application conditions. For example, is it applicable to all processes that can be modelled using deterministic ODE?

While the greybox models can in principle be used in any situation where a set of ODEs describe the phenomena at hand, the approach works best for reduced order models (possibly lumped) i.e. models where the deterministic part (drift term) of the stochastic differential equation describes only the most important phenomena, while the stochastic part (the diffusion term) then takes care of the deviations from the deterministic part, i.e. model approximations, measurement errors for the input/forcing variables and unrecognized input variables. Besides, there can be computational concerns in very high dimensional problems, such as weather systems, where estimating the likelihood for the model parameters is rather computationally intensive. This is now added to the discussions in the conclusion.

2. Figure S3 seems a little bit confusing. The authors are suggested to find a better diagram form and give more details about the figure.

As mentioned, we have removed the old Figure S3 showing the visited parts of $\Delta P - Q$ space in the randomization, because its size was not justified by the content it provided. Besides, the randomized values of the pressure and cross-flow can be seen from Figure S4a. Our new Figure S3 gives model predictions for constant pressure and constant cross-flow input.

REVIEWERS' COMMENTS:

Reviewer #1 (Remarks to the Author):

I have only one concern left. Should the "filtration" in title be specifically changed to "ultrafiltration"? Because word "filtration" includes many processes, particle filtration, microfiltration, ultrafiltration, hyper filtration (RO), the model developed in this study can only be valid for ultrafiltration. It is all up to the authors.

Reviewer #2 (Remarks to the Author):

The authors did an impressive work to better describe their experimental conditions. This article should be published in the present form. Also, the article bring an interesting new approach for membrane fouling modelling. I would be pleased to discuss with the authors as we work on similar problem but with a different statistical approach.

Reviewer #3 (Remarks to the Author):

Thanks to the authors for taking my comments into consideration. The manuscript is in good shape and ready for publication.
